# *Nod2* and *Nod2*-regulated microbiota protect BALB/c mice from diet-induced obesity and metabolic dysfunction

**DOI:** 10.1038/s41598-017-00484-2

**Published:** 2017-04-03

**Authors:** Ivan Rodriguez-Nunez, Tiffany Caluag, Kori Kirby, Charles N. Rudick, Roman Dziarski, Dipika Gupta

**Affiliations:** Indiana University School of Medicine–Northwest, Gary, IN 46408 USA

## Abstract

Genetics plays a central role in susceptibility to obesity and metabolic diseases. BALB/c mice are known to be resistant to high fat diet (HFD)-induced obesity, however the genetic cause remains unknown. We report that deletion of the innate immunity antibacterial gene *Nod2* abolishes this resistance, as *Nod2* ^−/−^ BALB/c mice developed HFD-dependent obesity and hallmark features of metabolic syndrome. *Nod2* ^−/−^ HFD mice developed hyperlipidemia, hyperglycemia, glucose intolerance, increased adiposity, and steatosis, with large lipid droplets in their hepatocytes. These changes were accompanied by increased expression of immune genes in adipose tissue and differential expression of genes for lipid metabolism, signaling, stress, transport, cell cycle, and development in both adipose tissue and liver. *Nod2* ^−/−^ HFD mice exhibited changes in the composition of the gut microbiota and long-term treatment with antibiotics abolished diet-dependent weight gain in *Nod2* ^−/−^ mice, but not in wild type mice. Furthermore, microbiota from *Nod2* ^−/−^ HFD mice transferred sensitivity to weight gain, steatosis, and hyperglycemia to wild type germ free mice. In summary, we have identified a novel role for *Nod2* in obesity and demonstrate that *Nod2* and *Nod2*-regulated microbiota protect BALB/c mice from diet-induced obesity and metabolic dysfunction.

## Introduction

The increasing prevalence of obesity is a major health concern worldwide^1^. Obesity is a risk factor for other diseases and more than half of all obese individuals develop metabolic abnormalities, which include hyperglycemia, hyperlipidemia, hepatic steatosis, and/or hypertension. Though the primary cause of obesity is an excess of caloric intake over expenditure, genetics and the environment play a central role in regulating metabolism and in the development of these diseases. In addition, the gut microbiota contributes to energy homeostasis in the host and changes in the composition of the gut microbiota can contribute to the development of obesity and metabolic disease^2, 3^. In turn, host genetics and environmental factors shape the microbiota and changes in either factor can alter intestinal bacterial populations. However, the genetic and environmental regulation of the microbiota is poorly understood and the specific changes in the gut bacteria that contribute to the development of obesity and metabolic disease are not identified.

Mouse genotype, similar to humans, greatly influences metabolism and different strains of mice differ in their response to high fat diet and sensitivity to metabolic diseases. BALB/c mice are naturally resistant to the development of high fat diet (HFD)-induced obesity, however the mechanism behind this resistance is not known^4^. There is increasing evidence for the role of innate immunity in the development of metabolic disease^5, 6^ and in this study we investigated the role of the pattern recognition receptor *Nod2* in the resistance of BALB/c mice to HFD-induced obesity.

*Nod2* is involved in innate immunity to bacteria and is expressed in pro-inflammatory and antigen presenting cells, such as monocytes and dendritic cells, and also in structural cells, such as hepatocytes, preadipocytes, and intestinal epithelial cells^7–10^. Nod2 is a cytoplasmic sensor for bacterial peptidoglycan fragments, and stimulation of Nod2 results in the activation of NF-κB and MAP kinase-signaling cascades and production of inflammatory molecules and anti-microbial peptides^11–13^. *Nod2* promotes a healthy microbiota in the intestine, and *Nod2*-deficient mice have an altered gut microbiota, which promotes colitis^14, 15^. *Nod2* was the first susceptibility gene identified for Crohn’s disease^16–18^, however, the functional role of *Nod2* in the pathogenesis of intestinal inflammation remains unknown and the role of *Nod2* in other inflammatory and non-inflammatory disorders is mostly unexplored^19–22^.

We demonstrate in this study that deletion of the pattern recognition receptor *Nod2* abolishes the resistance of BALB/c to HFD-induced obesity. *Nod2* ^−/−^ BALB/c mice on HFD gain substantially more weight than wild type (WT) BALB/c mice on HFD and become obese. Obese *Nod2* ^−/−^ BALB/c mice develop metabolic dysfunction including hyperlipidemia, hyperglycemia, and steatosis, and have increased adipose tissue mass and large lipid droplets in hepatocytes. *Nod2* ^−/−^ mice on HFD have dysbiosis and the altered gut microbiota contributes to the development of diet-dependent obesity and metabolic dysfunction.

## Results

### *Nod2* protects from diet-induced obesity in BALB/c mice

**Figure 1:**
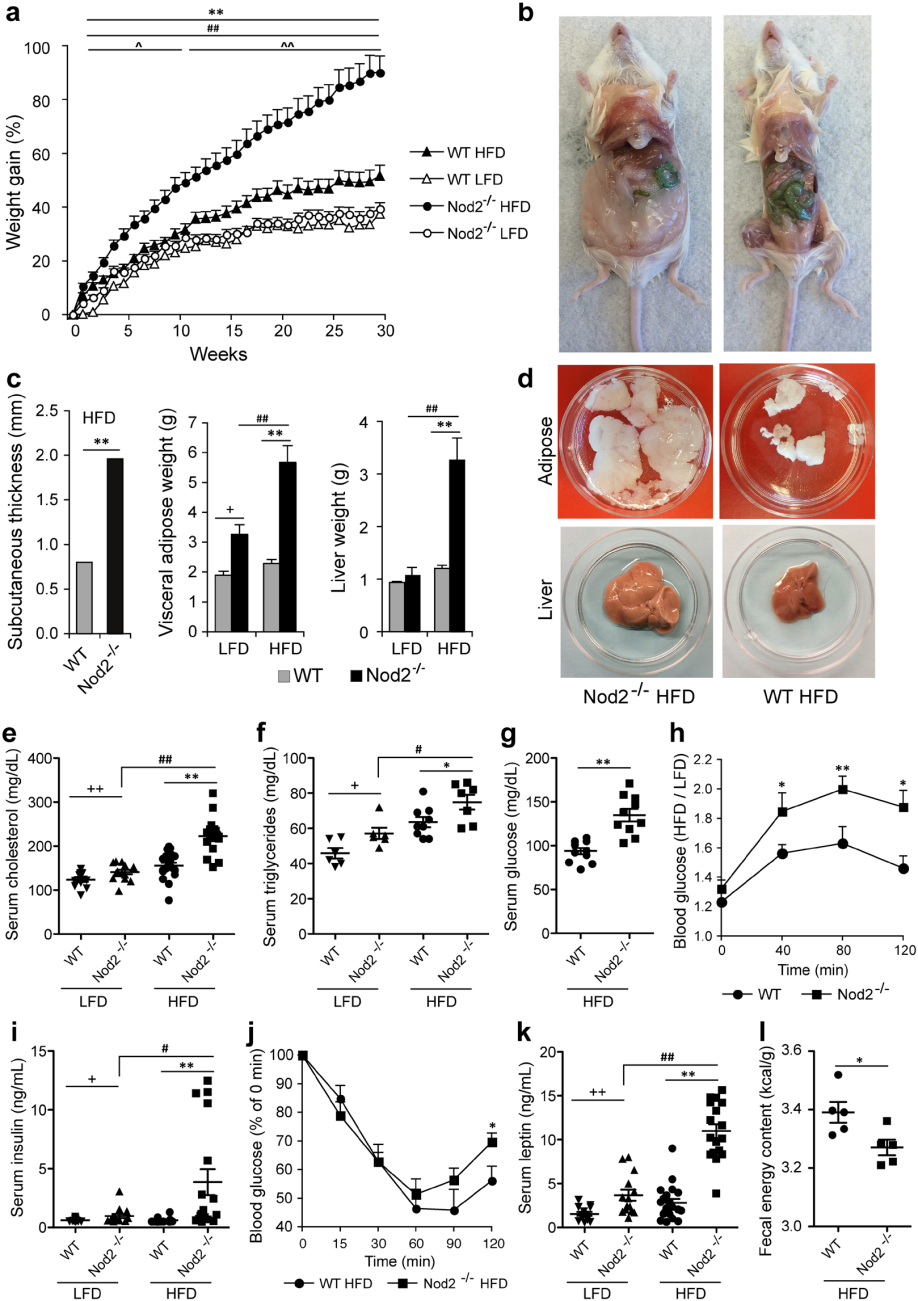
*Nod2* ^−/−^ mice on HFD become obese and develop hyperlipidemia, hyperglycemia, and glucose intolerance. WT and *Nod2* ^−/−^ mice were monitored for weight gain, increased adiposity, and assayed for metabolites. (**a**) Percent gain in body weight over week 0. (**b**–**d**) At week 30, (**b**) representative mice are shown for increase in overall size and visceral adipose tissue, (**c**) average increase in subcutaneous thickness and weight of visceral adipose tissue and liver per mouse, and (**d**) total visceral adipose tissue and liver from representative mice. (**e**–**j**) At week 30, mice were assayed for serum (**e**) cholesterol, (**f**) triglycerides, (**g**) glucose, (**i**) insulin, and (**k**) leptin, and for the development of (**h**) glucose tolerance and (**j**) insulin resistance. (**l**) Total caloric value in fecal samples. The results are (**a**) means ± SEM of 18–25 mice/group; (**c**,**h**,**j**) means ± SEM of 10–12 mice/group; or (**e**–**g**,**i**,**k**,**l**) for individual mice/group ± SEM. (**h**) Data for the glucose tolerance test is shown as ratio of HFD to LFD for each strain. *P ≤ 0.05, **P ≤ 0.001, *Nod2* ^−/−^ HFD *versus* WT HFD; ^#^ *P* ≤ 0.05, ^##^ *P* ≤ 0.001, *Nod2* ^−/−^ HFD *versus Nod2* ^−/−^ LFD; ^*P* ≤ 0.05, ^^*P* ≤ 0.001, WT HFD *versus* WT LFD; ^+^ *P* ≤ 0.05, ^++^ *P* ≤ 0.001, *Nod2* ^−/−^ LFD *versus* WT LFD. The results for food consumed and voluntary wheel activity are shown in Fig. S1.

WT and *Nod2* ^−/−^ female mice on BALB/c background, matched for weight (average 18 g) and age (6–7 weeks old) at the beginning of the experiment were maintained on HFD (fat 60 kcal%) or low fat diet (LFD, fat 10 kcal%) for 30 weeks. The total calories/g of food was the same for both diets. We observed a dramatic increase in weight gained by *Nod2* ^−/−^ mice compared with WT mice on HFD (Fig. 1a). *Nod2* ^−/−^ mice gained significantly more weight than WT mice starting from week 2 on HFD, and at week 30 *Nod2* ^−/−^ mice gained almost twice as much weight as WT mice. As expected, WT and *Nod2* ^−/−^ mice on HFD gained significantly more weight than WT and *Nod2* ^−/−^ mice on LFD. There was no difference in weight gained between WT and *Nod2* ^−/−^ mice on LFD. Our data with WT mice concur with previous data showing that WT BALB/c mice do not become obese on HFD^4^.

*Nod2* ^−/−^ mice on HFD were visibly larger with substantially more fat deposits than WT HFD mice (Fig. 1b) and had significantly increased subcutaneous and visceral adipose tissue and an enlarged and fatty liver compared with WT HFD mice (Fig. 1c and d). There was no difference in the amount of food consumed or water intake between WT and *Nod2* ^−/−^ HFD mice; average food consumed/mouse/day was 2.0 g for both genotypes and (Supplementary Fig. S1); the average water intake/mouse/day was 4.0 ml for both genotypes. There was also no significant difference in the average running distance/24 h between WT (9.48 km) and *Nod2* ^−/−^ (9.59 km) mice on regular chow (Supplementary Fig. S1).

Thus, our data demonstrate that in WT mice *Nod2* protects from the development of HFD-induced obesity and that *Nod2* deletion reverses the resistance to obesity observed in WT BALB/c mice. The increased weight gain in *Nod2* ^−/−^ HFD mice compared with WT HFD mice is not due to increased food consumption or due to an inherent lower activity.

### *Nod2* protects from diet-induced hyperlipidemia, hyperglycemia, and glucose intolerance in BALB/c mice

We next examined the effect of *Nod2* and HFD on lipid and carbohydrate metabolism. *Nod2* ^−/−^ HFD mice exhibited significantly higher levels of serum cholesterol and triglycerides compared with WT HFD mice and compared with *Nod2* ^−/−^ and WT mice on LFD (Fig. 1e and f). *Nod2* ^−/−^ HFD mice had significantly higher fasting glucose and impaired ability to restore blood glucose in response to a bolus of glucose compared with WT HFD mice (Fig. 1g and h), which indicates development of glucose intolerance. *Nod2* ^−/−^ HFD mice had significantly higher levels of serum insulin than WT HFD, *Nod2* ^−/−^ LFD, and WT LFD mice (Fig. 1i), and exhibited a mild but significantly reduced response to exogenous insulin compared with WT HFD mice 120 min after an insulin challenge (Fig. 1j), which indicates the development of insulin resistance and suggests that partial compensation may be achieved by increased insulin production. *Nod2* ^−/−^ HFD mice had significantly higher serum levels of the anorexic hormone leptin than WT HFD mice, *Nod2* ^−/−^ LFD mice, and WT LFD mice (Fig. 1k). WT HFD mice had significantly higher triglycerides than *Nod2* ^−/−^ LFD and WT LFD mice, which indicates the development of mild metabolic dysfunction in WT HFD mice. *Nod2* ^−/−^ LFD mice also had a smaller but significant increase in serum lipids, glucose, insulin, and leptin compared with WT LFD mice. Thus, our results demonstrate that *Nod2* ^−/−^ HFD mice develop hyperlipidemia, hyperglycemia, and glucose intolerance, which are hallmark manifestations of metabolic syndrome in humans.

To determine any differences in the total energy extracted from food between *Nod2* ^−/−^ HFD mice and WT HFD mice, we measured the total calories present in the stool samples of these mice. Our results show that total caloric value of *Nod2* ^−/−^ HFD feces was significantly lower than WT HFD feces (Fig. 1l). These results indicate that *Nod2* ^−/−^ mice on HFD extract more energy from the same amount of food than WT mice on HFD, which may be due to differences in the metabolism of host or gut microbiota.

### *Nod2* protects from diet-induced histopathology in BALB/c mice

**Figure 2:**
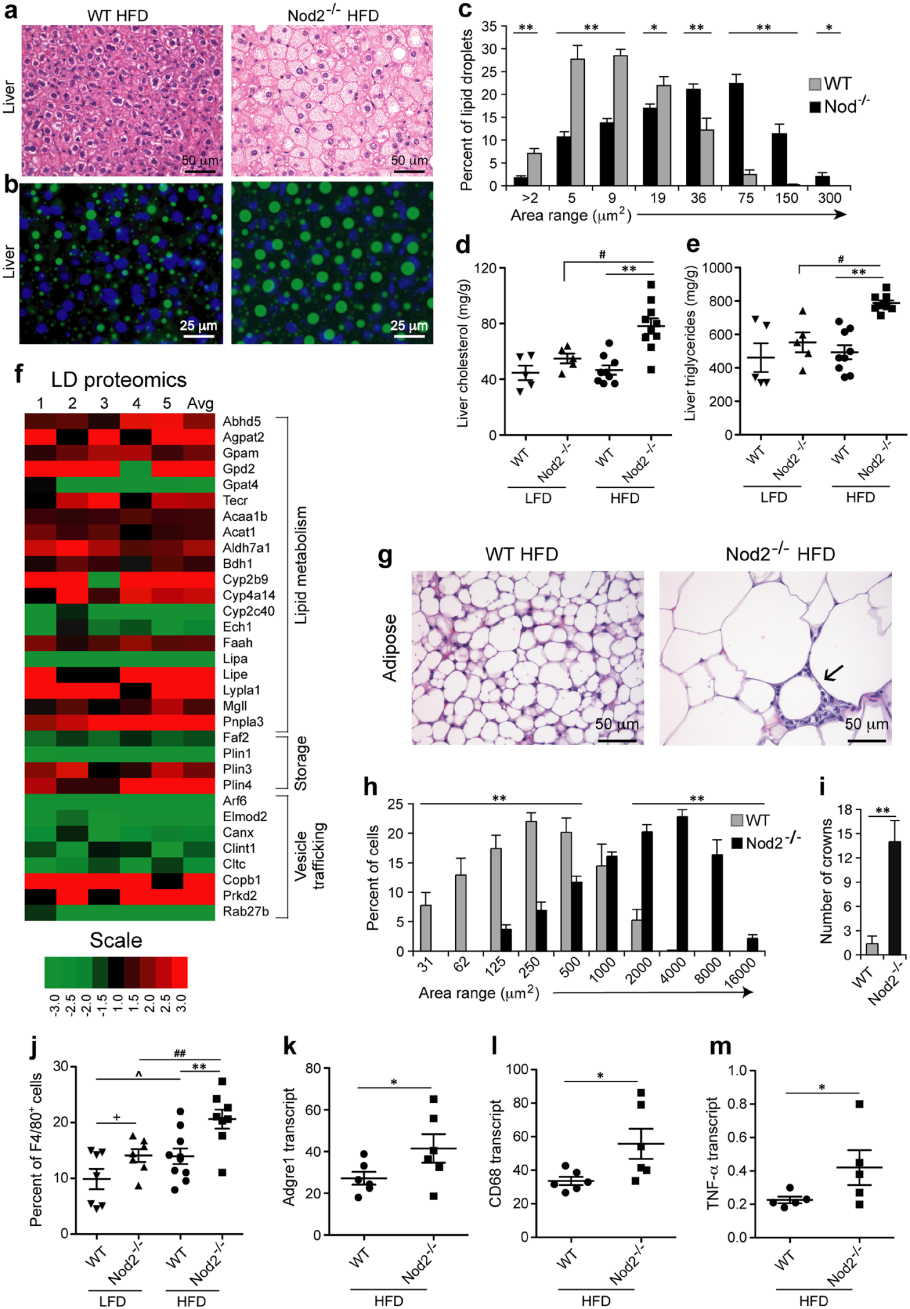
*Nod2* ^−/−^ mice on HFD develop steatosis and have large stressed adipocytes with increased infiltration of macrophages. (**a**,**b**) Liver sections from *Nod2* ^−/−^ and WT mice stained with (**a**) H&E or (**b**) BODIPY and DAPI. (**c**) Percent frequency distribution of the area (µm^2^) of ~5,000 LDs/group. *(* **d**,**e**) Quantification of liver cholesterol and triglycerides. (**f**) Heatmap representation of the fold increase (red) or decrease (green) in the abundance of proteins present in LD-enriched fraction from liver. The fold ratio for individual *Nod2* ^−/−^ HFD mice to the average of WT HFD mice were computed (lanes 1 to 5) with the average of the fold for all *Nod2* ^−/−^ HFD mice. (**g**) Adipose tissue H&E sections and a dying cell (→) is indicated. (**h**) Percent frequency distribution the area of ~8,000 adipocytes/group. (**i**) Average numbers of dying adipocytes (crowns) per 3,000 cells in H&E stained sections. (**j**) Quantification of CD45^+^F4/80^+^ macrophage in adipose tissue. (**k**–**m**) Transcript levels for *Adgre1*, *Cd68*, and *Tnfα* in adipose tissue. Images are representative and results are (**c**,**h**,**i**) means ± SEM of 6 mice/group and (**d**,**e**,**j**–**m**) for individual mice with *N* = 5–10 mice/group. *P ≤ 0.05, **P ≤ 0.001, *Nod2* ^−/−^ HFD *versus* WT HFD; ^#^ *P* ≤ 0.05, ^##^ *P* ≤ 0.001, *Nod2* ^−/−^ HFD *versus Nod2* ^−/−^ LFD; ^*P* ≤ 0.05, WT HFD *versus* WT LFD; and ^+^ *P* ≤ 0.05, *Nod2* ^−/−^ LFD *versus* WT LFD.

We characterized the histopathology in the liver and adipose tissue of *Nod2* ^−/−^ obese mice. *Nod2* ^−/−^ HFD mice had significantly larger hepatocytes than WT mice on HFD and the majority of these cells had a foamy appearance, which indicates lipid accumulation (Fig. 2a). In BODIPY stained sections there was a dramatic and significant increase in the size of lipid droplets (LDs) in *Nod2* ^−/−^ mice compared with WT mice (Fig. 2b). The area for LDs ranged from 2 μm^2^ to 300 μm^2^ with the highest percentage of LDs in the 36 to 75 μm^2^ range for *Nod2* ^−/−^ mice and 5 to 9 μm^2^ range for WT mice (Fig. 2c). *Nod2* ^−/−^ mice on HFD had significantly higher levels of liver cholesterol and triglycerides than WT HFD, *Nod2* ^−/−^ LFD, and WT LFD mice (Fig. 2d and e). Thus, our histological and biochemical data further support our results showing development of obesity in *Nod2* ^−/−^ HFD mice and also support our data for WT mice, which do not become obese and do not have a fatty liver (Fig. 1).

We next identified differences in the proteins associated with LDs between *Nod2* ^−/−^ and WT mice on HFD. We isolated proteins from LD-enriched fractions and identified them by mass spectrometry. All proteins that were significantly different (*P* ≤ 0.05 and fold change ≥1.5 or ≤0.6) between *Nod2* ^−/−^ and WT mice are directly or indirectly involved in lipid metabolism or in vesicle trafficking (Fig. 2f). *Nod2* ^−/−^ HFD mice compared with WT HFD mice had significantly higher levels of Abhd5, Agpat2, Gpam, and Gpd2 but decreased levels of Gpat4, proteins associated with biosynthesis of glycerolipids. Tecr, a protein involved in the synthesis of very long chain fatty acids was increased in *Nod2* ^−/−^ HFD mice. Many proteins that participate in the catabolism of lipids were also more abundant in *Nod2* ^−/−^ HFD mice than in WT HFD mice, including Acaa1b, Acat1, Aldh7a1, Bdh1, Cyp2b9, Cyp4a14, Faah, Lipe, Lypla1, Mgll, and Pnpla3. Proteins associated with lipid storage were selectively up regulated or down regulated, Plin3 and Plin4 were present at higher and Faf2 and Plin1 at lower levels in *Nod2* ^−/−^ HFD mice. Several proteins involved in vesicle trafficking were decreased in the LD-enriched fraction of *Nod2* ^−/−^ HFD mice compared with WT HFD mice. However, Copb1, a protein associated with non-clathrin coated vesicles with a role in limiting lipid storage, was significantly higher in *Nod2* ^−/−^ HFD mice than WT HFD mice. These data indicate that the larger LDs in *Nod2* ^−/−^ HFD mice compared with WT HFD mice are accompanied by changes in lipid metabolizing enzymes and in vesicle trafficking proteins.

*Nod2* ^−/−^ mice on HFD had enlarged adipocytes with significantly larger area than WT HFD mice (Fig. 2g and h). The area for adipocytes ranged from 31 μm^2^ to 16,000 μm^2^ with the highest percentage of cells in the 2000 to 4000 μm^2^ range for *Nod2* ^−/−^ mice and 250 to 500 μm^2^ for WT mice (Fig. 2h). *Nod2* ^−/−^ mice had significantly more “crowns”, which are dead adipocytes surrounded by immune cells (Fig. 2g and i, indicated by an arrow) and are a characteristic feature of stressed adipose tissue associated with obesity. *Nod2* ^−/−^ mice on HFD had significantly more F4/80^+^ macrophages in adipose tissue than WT mice on HFD, *Nod2* ^−/−^ LFD, and WT LFD mice (Fig. 2j) with a corresponding increase in the transcripts for *Adgre1* (gene for F4/80) and *Cd68*, another marker for monocytes/macrophages (Fig. 2k and l). Expression of *Tnfα*, an inflammatory cytokine associated with obesity was enhanced (Fig. 2m). Thus, our data demonstrate that *Nod2* protects from lipid accumulation in the adipose tissue and liver, and that development of diet-dependent obesity in *Nod2* ^−/−^ HFD mice is associated with steatosis, formation of large LDs in hepatocytes, stressed adipocytes, and infiltration of macrophages in the adipose tissue.

### Diet-dependent obesity in *Nod2*^−/−^ mice is associated with changes in the expression of adipose tissue genes involved in immune responses and intermediary metabolism

**Figure 3:**
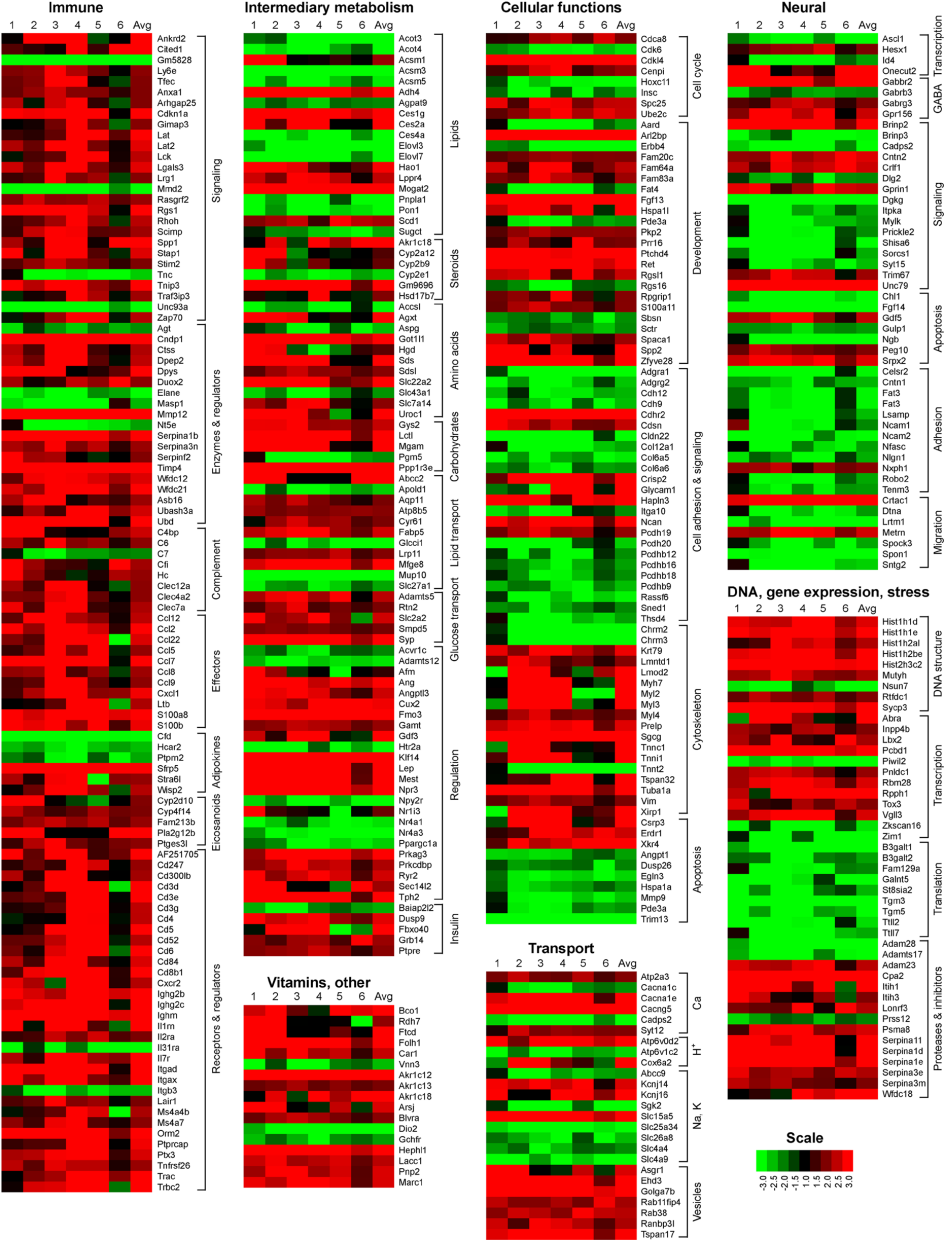
Differential expression of genes for immunity, metabolism, and other cellular functions in the adipose tissue of *Nod2* ^−/−^ mice on HFD. Heatmap representation of the fold increase (red) or decrease (green) for gene expression in the adipose tissue of *Nod2* ^−/−^ HFD mice compared with WT HFD mice. The genes are grouped based on function or pathway. The fold ratio for individual *Nod2* ^−/−^ HFD mice to the average of WT HFD mice was computed and is shown (lanes 1 to 6) with the average of the fold for all *Nod2* ^−/−^ HFD mice. Genes that had a fold change of ≥2 or ≤0.5 (≤−2) and *P* ≤ 0.05 with 5% FDR are included in the heatmap. *N* = 6 mice/group. The numerical data for fold increase in individual mice, the average, *P* value, and FDR are shown in Supplementary Table S1.

We identified genes that were differentially expressed in the visceral adipose tissue of WT and *Nod2* ^−/−^ BALB/c mice maintained on HFD for 30 weeks using RNA transcriptomics. *Nod2* ^−/−^ HFD mice had 357 genes with significantly increased expression (and fold change ≥2) and 169 genes with significantly decreased expression (and fold change ≤0.5) compared with WT mice on HFD (Supplementary Fig. S2). 79% of the differentially expressed genes are associated with a function or pathway (Ensembl or DAVID) and are shown in Fig. 3 and Supplementary Table S1. More than 100 genes that directly or indirectly participate in immune responses were differentially regulated and the majority of these genes (>80) were significantly up regulated in the adipose tissue of *Nod2* ^−/−^ HFD mice compared with WT HFD mice. These genes code for transcription factors, signaling molecules, effector molecules, enzymes, and receptors, all involved in regulating immune cells and responses. For example, *Nod2* ^−/−^ mice on HFD had increased expression of genes for (i) chemokines *Ccl2*, *Ccl5*, *Ccl7*, *Ccl8*, *Ccl12*, *Ccl22*, and *Cxcl1*, which recruit monocytes, eosinophils, and lymphocytes; (ii) enzymes involved in arachidonic acid and eicosanoid metabolism, *Cyp2c39*, *Cyp2d10*, *Cyp4f14*, *Fam213b*, *Pla2g12b*, and *Ptges3l*; (iii) receptors involved in T cell activation, including *Cd3δ*, *Cd3ε*, *Cd3γ*, *Cd4*, *Cd5*, *Cd6*, and *Cd52*; and (iv) adipokines *Sfrp5*, *Stra6l*, and *Wisp2*, and the protease *Cpn1*. Expression of genes for adipsin (*Cfd*) and regulators of adipokines, *Hcar2* and *Ptprn2*, was decreased.

The second largest group of genes that was differentially regulated between WT and *Nod2* ^−/−^ HFD mice directly or indirectly participates in intermediary metabolism, primarily lipid metabolism (Fig. 3, Supplementary Table S1). These genes include: (i) enzymes involved in breakdown of fatty acids and glycerolipids, for example, *Acot3*, *Acot4*, *Ces4a*, *Agpat9*, and *Pnpla1* were increased; (ii) members of the acyl-CoA synthetase medium chain family were up regulated (*Acsm1*) or down regulated (*Acsm3* and *Acsm5*); (iii) enzymes in the synthesis of fatty acids and triglycerides, *Mogat2* and *Scd1*, were increased; (iv) transporters for fatty acids, cholesterol, glycerolipids, and glycerol, *Abcc2*, *Aqp11*, *Atp8b5*, *Fabp5*, and *Mfge8*, were up regulated, and *Apold1*, *Glcci1*, and *Slc27a1*, were down regulated.

*Nod2* ^−/−^ mice on HFD had several differentially expressed genes that directly or indirectly participate in amino acid and carbohydrate metabolism compared with WT HFD mice (Fig. 3, Supplementary Table S1). These genes include: (i) enzymes in amino acid metabolism, *Accsl*, *Agxt*, and *Aspg* were decreased or *Got1l1*, *Hgd*, *Sds*, and *Sdsl* were increased and (ii) amino acid transport, *Slc22a2*, *Slc7a14*, and *Uroc1* were increased. Genes for: (i) enzymes in carbohydrate metabolism, *Gys2*, *Lctl*, *Mgam*, and *Ppp1r3e* and (ii) glucose transport, *Adamts5*, *Rtn2*, *Slc2a2*, *Smpd5*, and *Syp* were up regulated, but (iii) insulin signaling, *Dusp9*, *Fbxo40*, *Grb14*, and *Ptpre* were down regulated.

*Nod2* ^−/−^ mice on HFD had an overall decrease in the expression of genes involved in (i) neural signaling, including *Brinp3*, *Cadps2*, *Dlg2*, *Itpka*, *Mylka*, *Prickle2*, *Shisa6*, *Sorcs1*, *Syt15*; (2) nerve adhesion, *Celsr2*, *Cntn1*, *Fat3*, *Lsamp*, *Ncam1*, *Ncam2*, *Nfasc*, and *Nlgn1*; and (iii) transcriptional regulators for neural development, *Ascl1*, *Hesx1*, and *Id4* (Fig. 3, Supplementary Table S1).

Genes involved in other cellular functions, including cell cycle, development, cell adhesion, and cytoskeleton were also differentially expressed (Fig. 3, Supplementary Table S1). *Nod2* ^−/−^ HFD mice also had an overall decrease in the expression of genes involved in (i) cell adhesion and signaling, including *Adgra1*, *Adgrg2*, *Cdh12*, *Cdh9*, *Cldn22*, *Col12a1*, *Col6a5*, *Col6a6*, *Pcdh20*, *Pcdhb12*, *Pcdhb16*, *Pcdhb18*, *Pcdhb9*, *Rassf6*, *Sned1*, and *Thsd4*; and (ii) inhibition of apoptosis, including *Angpt1*, *Dusp26*, *Egln3*, *Hspa1a*, and *Pde3a*. There was an overall increase in expression of genes involved in: (i) DNA structure, repair and chromatin silencing, for example, *Hist1h1d*, *Hist1h1e*, *Hist1h2al*, *Hist1h2be*, *Hist2h3c2*, and *Mutyh*; (ii) transcription and post-transcriptional processing, *Abra*, *Inpp4b*, *Lbx2*, *Pcbd1*, *Pnldc1*, *Rbm28*, and *Rpph1*; and (iii) proteases and protease inhibitors, *Adam23*, *Cpa2*, *Itih1*, *Itih3*, *Lonrf3*, *Psma8*, *Serpina11*, *Serpina1d*, *Serpina1e*, *Serpina3e-ps*, *Serpina3m*, and *Wfdc18*. However, there was a noticeable decrease in genes involved in translation and post-translational processing, *B3galt1*, *B3galt2*, *Fam129a*, *Galnt5*, *St8sia2*, *Tgm3*, *Tgm5*, *Ttll2*, and *Ttll7*.

Thus, our results indicate that genes involved in a wide array of pathways and cellular functions are differentially regulated in the adipose tissue of *Nod2* ^−/−^ HFD mice compared with WT HFD mice. These changes in gene expression may contribute to the increased adiposity, steatosis, obesity, and metabolic dysfunction observed in *Nod2* ^−/−^ mice on HFD. Future studies should confirm these changes at the protein level and focus on their role in the development of obesity in *Nod2* ^−/−^ HFD mice.

### Diet-dependent obesity in *Nod2*^−/−^ mice is associated with changes in the expression of liver genes involved in intermediary metabolism

**Figure 4:**
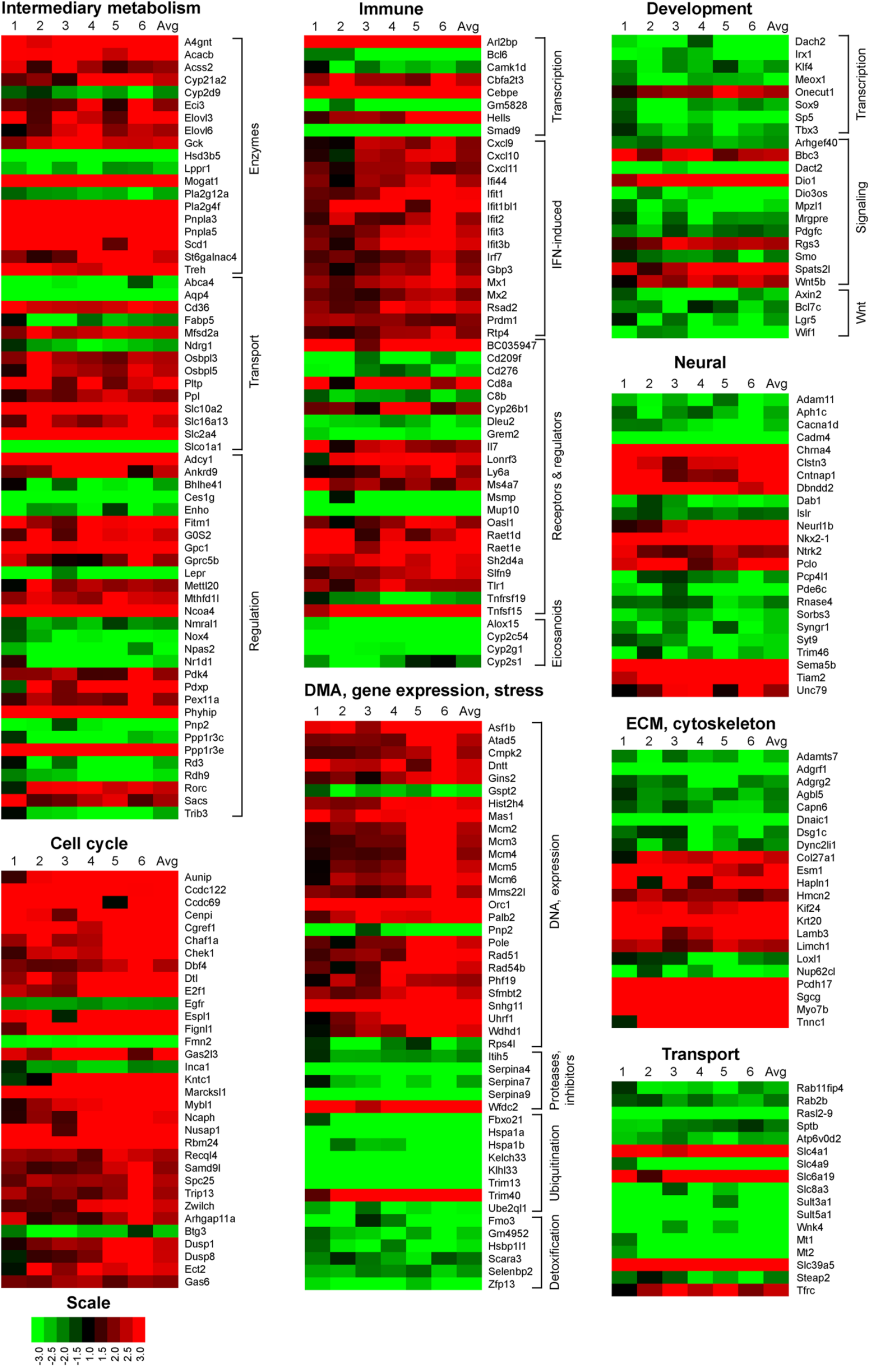
Differential expression of genes for immunity, metabolism, and other cellular functions in the liver of *Nod2* ^−/−^ mice on HFD. Heatmap representation of the fold increase (red) or decrease (green) for gene expression in the adipose tissue of *Nod2* ^−/−^ HFD mice compared with WT HFD mice. The genes are grouped based on function or pathway. The fold ratio for individual *Nod2* ^−/−^ HFD mice to the average of WT HFD mice was computed and is shown (lanes 1 to 6) with the average of the fold for all *Nod2* ^−/−^ HFD mice. Genes that had a fold change of ≥2 or ≤0.5 (≤−2) and *P* ≤ 0.05 with 5% FDR are included in the heatmap. *N* = 6 mice/group. The numerical data for fold increase in individual mice, the average, *P* value, and FDR are shown in Supplementary Table S2.

We next identified genes that were differentially expressed in the liver of WT and *Nod2* ^−/−^ BALB/c mice maintained on HFD for 30 weeks using RNA transcriptomics. *Nod2* ^−/−^ HFD mice had 188 genes with significantly increased expression (and fold change ≥2) and 130 genes with significantly decreased expression (and fold change ≤0.5) compared with WT mice on HFD (Supplementary Fig. S2). 87% of the differentially expressed genes are associated with a function or pathway (Ensembl or DAVID) and are shown in Fig. 4 and Supplementary Table S2. Of these genes, there were more than 60 genes that directly or indirectly participate in intermediary metabolism, primarily lipid metabolism and include genes for enzymes, transport, and regulation. For example, *Nod2* ^−/−^ HFD mice had higher expression of enzymes and regulators involved in: (i) synthesis and storage of fatty acids, triglycerides, steroids, and glycolipids, *Acacb*, *Acss2*, *Elovl3*, *Elovl6*, *Fitm1*, *G0s2*, *Hsd3b5*, *Mogat1*, *Scd1*, and *St6galnac4*; (ii) catabolism of lipids, *Pla2g4f*, *Pnpla3*, *Pnpla5*, *Pex11a*, and *Phyhip*; and (iii) carbohydrate metabolism, *Gck*, *Pdk4*, and *Ppp1r3e*. Several genes involved in transport of metabolites and nutrients were down regulated, including *Aqp4*, *Fabp5*, *Ndgr1*, and *Slco1A1*, whereas other transporters were up regulated, including *Cd36*, *Mfsd2a*, *Osbpl5*, *Pltp*, *Slc10a2*, *Slc16a13*, and *Slc2a4 (Glut4)*. *Nod2* ^−/−^ HFD mice had decreased expression of the leptin receptor (*Lepr*).

*Nod2* ^−/−^ mice on HFD had about 50 genes involved in immune responses that were differentially expressed in the liver compared with WT mice on HFD (Fig. 4, Supplementary Table S2). Transcription factors involved in the activation of immune cells, *Arl2bp*, *Cbfa2t3*, and *Cebpε*, were increased, whereas transcriptional repressors *Bcl6* and *Cam1kd* were decreased. There was a noticeable increase in the expression of many interferon-induced genes, including the chemokines *Cxcl9*, *Cxcl10*, and *Cxcl11*. Genes for the T cell receptor *Cd8α* and retinoic acid metabolism, *Cyp26b1*, *Raet1δ*, and *Raet1*ε, that regulate T and NK cell immune responses were induced, but genes in arachidonic acid metabolism, *Alox15*, *Cyp2c54*, *Cyp2g1*, and *Cyp2s1*, were inhibited.

*Nod2* ^−/−^ mice on HFD had increased expression of many liver genes involved in DNA structure, replication, and repair (Fig. 4, Supplementary Table S2). Genes for serine protease inhibitors (*Itih5*, *Serpina4-ps1*, *Serpina7*, and *Serpina9*), chaperones (*Hsp1a* and *Hsp1b*), and ubiquitination (*Kelch33*, *Klhl33*, *Trim13*, and *Ube2ql1*) were decreased. Genes for cell cycle and cell proliferation were overwhelmingly induced, however many genes involved in development were down regulated, including transcription factors (*Irx1*, *Klf4*, *Meox1*, *Sox9*, *Sp5*, and *Tbx3*), signaling molecules (*Arhgef40*, *Dact2*, *Mpzl1*, *Mrgpre*, and *Pdgfc*) and components of the Wnt pathway (*Axin2*, *Bcl7c*, *Wif1*, and *Lgr5*). Several genes involved in neural development were down regulated, including proteins involved in ion and vesicular transport (*Adam11*, *Cacnα1d*, *Syngr1*, and *Syt9*) and neural signaling (*Aph1c*, *Cadm4*, and *Pde6c*). However, genes for acetylcholine receptor (*Chrna4*) and signaling at the neuronal junction were up regulated (*Clstn3*, *Cntnap1*, and *Pclo*). The tyrosine kinase receptor, *Ntrk2*, which has a role in suppressing appetite, was also up regulated (Fig. 4, Supplementary Table S2).

*Nod2* ^−/−^ mice on HFD had increased expression of many genes involved in extracellular matrix organization and cell adhesion, including *Col27a1*, *Esm1*, *Hapln1*, *Hmcn2*, *Lamb3*, *Myo7b*, and *Pcdh17*. Genes involved in vesicle trafficking (*Rab11fip4*, *Rab2b*, *Rasl2–9*, and *Sptb*) and proton, sodium and sulfur transport (*Atp6v0d2*, *Slc4a9*, *Slc8a3*, *Sult3a1*, *Sult5a1*, and *Wnk4*) were down regulated, whereas genes for zinc and iron uptake (*Slc39a5* and *Tfrc*) were increased (Fig. 4, Supplementary Table S2).

These results indicate that in the liver, genes involved in a wide array of pathways and cellular functions are differentially regulated by HFD in *Nod2* ^−/−^ mice compared with WT mice. These changes in gene expression may contribute to the increased adiposity, steatosis, obesity, and metabolic dysfunction observed in *Nod2* ^−/−^ mice on HFD. Future studies should confirm these changes at the protein level and focus on their role in the development of obesity in *Nod2* ^−/−^ HFD mice.

### *Nod2*^−/−^ genotype and HFD and LFD affect diversity of stool microbiome

Because intestinal microbiome is important for energy extraction from food, we determined the diversity of intestinal bacteria in WT and *Nod2* ^−/−^ mice on HFD and LFD, to identify in detail the differences in their intestinal microbiota. We isolated DNA from stool microbiota and performed genetic phylotyping (community profiling) using pyrosequencing of the variable regions of bacterial 16S ribosomal RNA (rRNA) genes. To determine the effect of genotype, we compared microbiomes in WT and *Nod2* ^−/−^ mice on the same diets, and to determine the effect of diets, we compared microbiomes in mice of the same genotype fed HFD or LFD.

We first evaluated α-diversity of the microbiomes of WT and *Nod2* ^−/−^ mice on HFD and LFD. We identified a total of 223 species and 4425 operational taxonomic units (OTUs) in the stools of all mice: 153, 172, 180, and 164 species and 2344, 2589, 2392, and 2381 OTUs in WT HFD, *Nod2* ^−/−^ HFD, WT LFD, and *Nod2* ^−/−^ LFD mice, respectively. The total number of OTUs identified in *Nod2* ^−/−^ HFD mice was significantly higher than in WT HFD mice, whereas the total numbers of OTUs in *Nod2* ^−/−^ LFD and WT LFD mice, as well as total numbers of species in all groups of mice were similar with no significant differences between the groups (Supplementary Fig. S3). The numbers of species and OTUs per mouse (microbiome richness) were similar in all groups of mice, except for WT LFD mice, which had significantly more species/mouse than WT HFD mice. Both Shannon diversity indices (H) and Shannon equitability indices (E_H_, reflecting microbiome evenness) were significantly higher for the species in *Nod2* ^−/−^ HFD than in WT HFD mice, and for both the species and OTUs in WT LFD than in *Nod2* ^−/−^ LFD mice, and also for both the species and OTUs in WT LFD than in WT HFD mice (Supplementary Fig. S3). These results show that both *Nod2* ^−/−^ genotype on HFD and WT genotype on LFD may increase α-diversity, although these changes are very modest.

**Figure 5:**
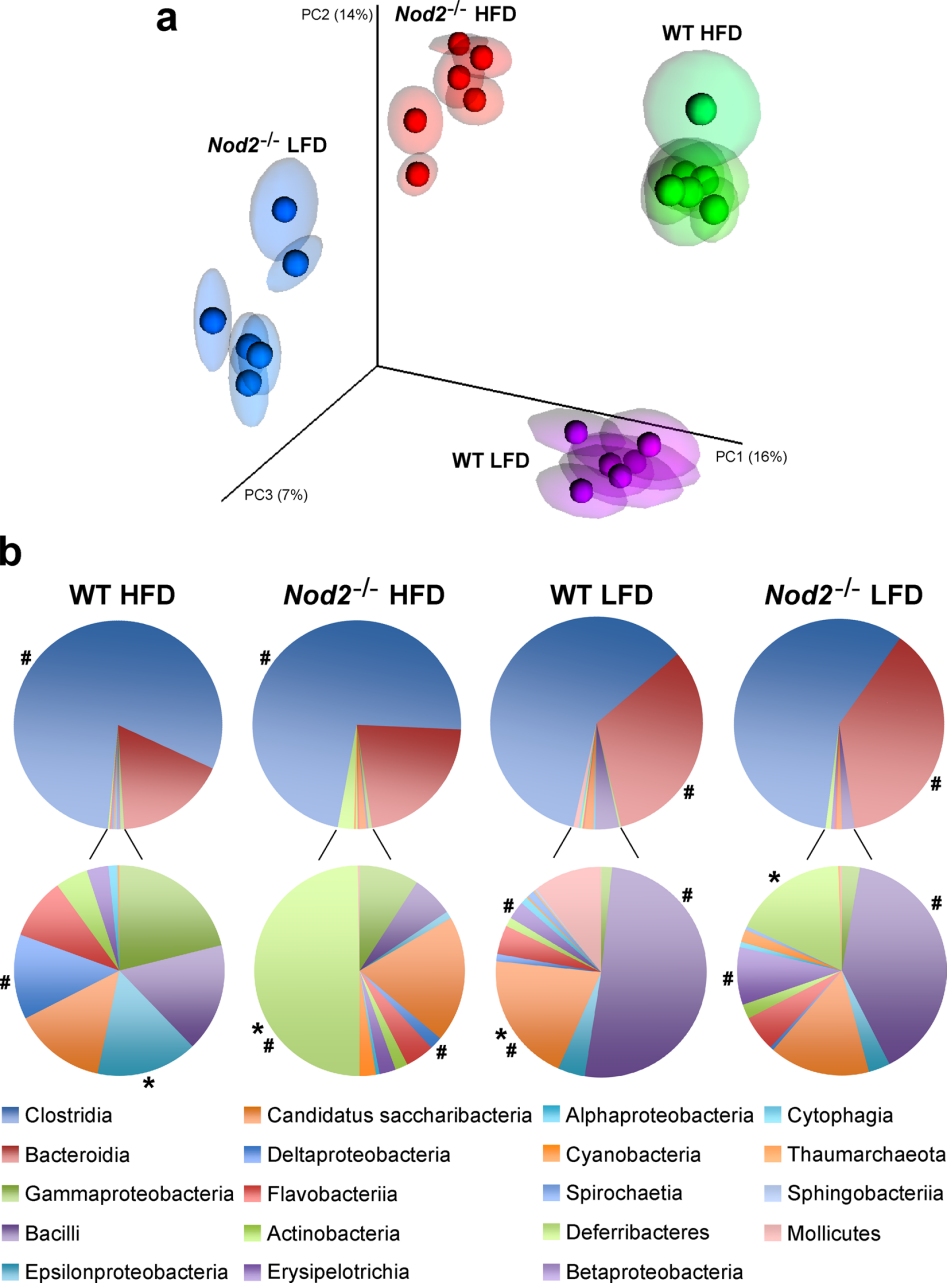
β-diversity in stool microbiota of WT HFD, *Nod2* ^−/−^ HFD, WT LFD, and *Nod2* ^−/−^ LFD mice. (**a**) Principal Coordinate Analysis (PCoA) by UniFrac (unweighted) of microbiomes. Each sphere with confidence elipsoid corresponds to a stool microbiome from one mouse. *N* = 6 mice/group. (**b**) Class abundance expressed as % of total stool microbiota; *classes with significantly (at *P* ≤ 0.05) increased abundance in WT *versus Nod2* ^−/−^ mice (on HFD or LFD); ^#^classes with significantly (at *P* ≤ 0.05) increased abundance in HFD *versus* LFD groups (for WT or *Nod2* ^−/−^ mice); *N* = 6 mice/group. The results for individual mice are shown in Supplementary Fig. S4. The entire microbiome analysis data comparing bacterial diversity in the stools of WT and *Nod2* ^−/−^ mice maintained on HFD and LFD have been deposited in NCBI SRA, accession No. SRP076031.

We then evaluated β-diversity of the microbiomes. Microbiota from stools of all four groups of mice (WT HFD, *Nod2* ^−/−^ HFD, WT LFD and *Nod2* ^−/−^ LFD) separated from each other in the Principal Coordinate Analysis (PCoA), with microbiota from each group of mice showing distinct and significant separation from the microbiota of all other groups of mice (Fig. 5a). These results suggested differences in β-diversity in the intestinal microbiomes between all four groups of mice with both the genotype (WT and *Nod2* ^−/−^) and the diet (HFD and LFD) affecting β-diversity of stool microbiomes. These results prompted us to further identify the differences between these microbiomes.

We detected many statistically significant effects of both *Nod2* genotype and the diet on bacterial β-diversity, with many significant changes in the abundance of various bacterial groups in the stools at all taxonomic levels. For example, at the class level (Fig. 5b and Supplementary Fig. S4), WT HFD mice had significantly increased abundance of *Epsilonproteobacteria* compared with *Nod2* ^−/−^ HFD mice, and *Nod2* ^−/−^ HFD mice had significantly increased abundance of *Deferribacteres* compared with WT HFD mice. In mice on LFD, WT mice had significantly higher abundance of *Candidatus saccharibacteria* than *Nod2* ^−/−^ mice, and *Nod2* ^−/−^ mice had significantly higher abundance of *Deferribacteres* than WT mice. The diet also had a pronounced effect on stool microbiota. HFD significantly increased the abundance of *Clostridia* and *Deltaproteobacteria* in both WT and *Nod2* ^−/−^ mice and also *Deferribacteres* in *Nod2* ^−/−^ mice, whereas LFD significantly increased the abundance of *Bacteroida*, *Bacilli*, and *Erysipelotrichia* in both WT and *Nod2* ^−/−^ mice, and also *Candidatus saccharibacteria* in WT mice.

At other taxonomic levels, significant differences in abundance in the following numbers of orders, families, genera, species, and OTUs (out of total of 27, 47, 109, 223, and 4425) were detected: between WT HFD and *Nod2* ^−/−^ HFD mice, 2, 2, 9, 26, and 548; between WT LFD and *Nod2* ^−/−^ LFD mice, 6, 10, 20, 46, and 601; between WT HFD and WT LFD, 8, 14, 24, 48, and 535; and between *Nod2* ^−/−^ HFD and *Nod2* ^−/−^ LFD mice, 6, 9, 16, 35, and 288, respectively (NCBI SRA, accession No. SRP076031). Thus, both *Nod2* genotype of mice and their diet together significantly influence the diversity of their microbiomes.

We then further analyzed the differences in microbiomes between *Nod2* ^−/−^ HFD mice and the remaining groups of mice, because these mice had significantly higher weight gain and significant manifestations of metabolic syndrome compared with the other groups of mice, and moreover, these differences were abolished by treatment with antibiotics (next section). *Nod2* ^−/−^ HFD mice, compared with WT HFD mice, had significantly higher abundance of six *Firmicutes*, three *Bacteroidetes*, two *Deferribacteres*, one *Proteobacteria*, and one *Actinobacteria* species (Table 1). Importantly, three of these bacterial species, both *Deferribacteres* and one *Firmicutes* (*Lachnoclostridium phytofermentans*) had significantly higher abundance in *Nod2* ^−/−^ HFD mice than in the other three groups of mice, thus showing the best correlation with obesity and metabolic syndrome, since *Nod2* ^−/−^ HFD mice was the only group with significantly higher obesity and metabolic syndrome. There were no bacterial species with significantly decreased abundance in *Nod2* ^−/−^ HFD mice compared with all other groups of mice.

**Table 1:** Bacterial species with significantly higher abundance in *Nod2* ^−/−^ HFD mice than in WT HFD mice*.

Phylum	Species	*Nod2* ^−/−^ HFD (% abundance)	WT HFD (% abundance)
Firmicutes	Hydrogenoanaerobacterium sp.	0.76	0.52
	Coprococcus eutactus	0.34	0.052
	Eubacterium rectale	0.18	0.049
	Lachnoclostridium (Clostridium) phytofermentans	0.15	0.0000
	Coprococcus catus	0.076	0.0000
	Blautia (Ruminococcus) obeum	0.027	0.0025
Bacteroidetes	Rikenella sp.	4.49	1.49
	Bacteroides sp.	1.58	0.71
	Butyricimonas sp.	0.011	0.0000
Deferribacteres	Mucispirillum sp.	2.27	0.0000
	Mucispirillum (Flexistipes) sp.	0.30	0.0000
Proteobacteria	Helicobacter ganmani	0.058	0.0000
Actinobacteria	Adlercreutzia equolifaciens	0.028	0.0025

*P < 0.05 for all species. The entire microbiome analysis data comparing bacterial diversity in the stools of WT and *Nod2* ^−/−^ mice maintained on HFD and LFD have been deposited in NCBI SRA, accession No. SRP076031.

### Diet-induced obesity in *Nod2*^−/−^ BALB/c mice is abolished by long-term antibiotic treatment

**Figure 6:**
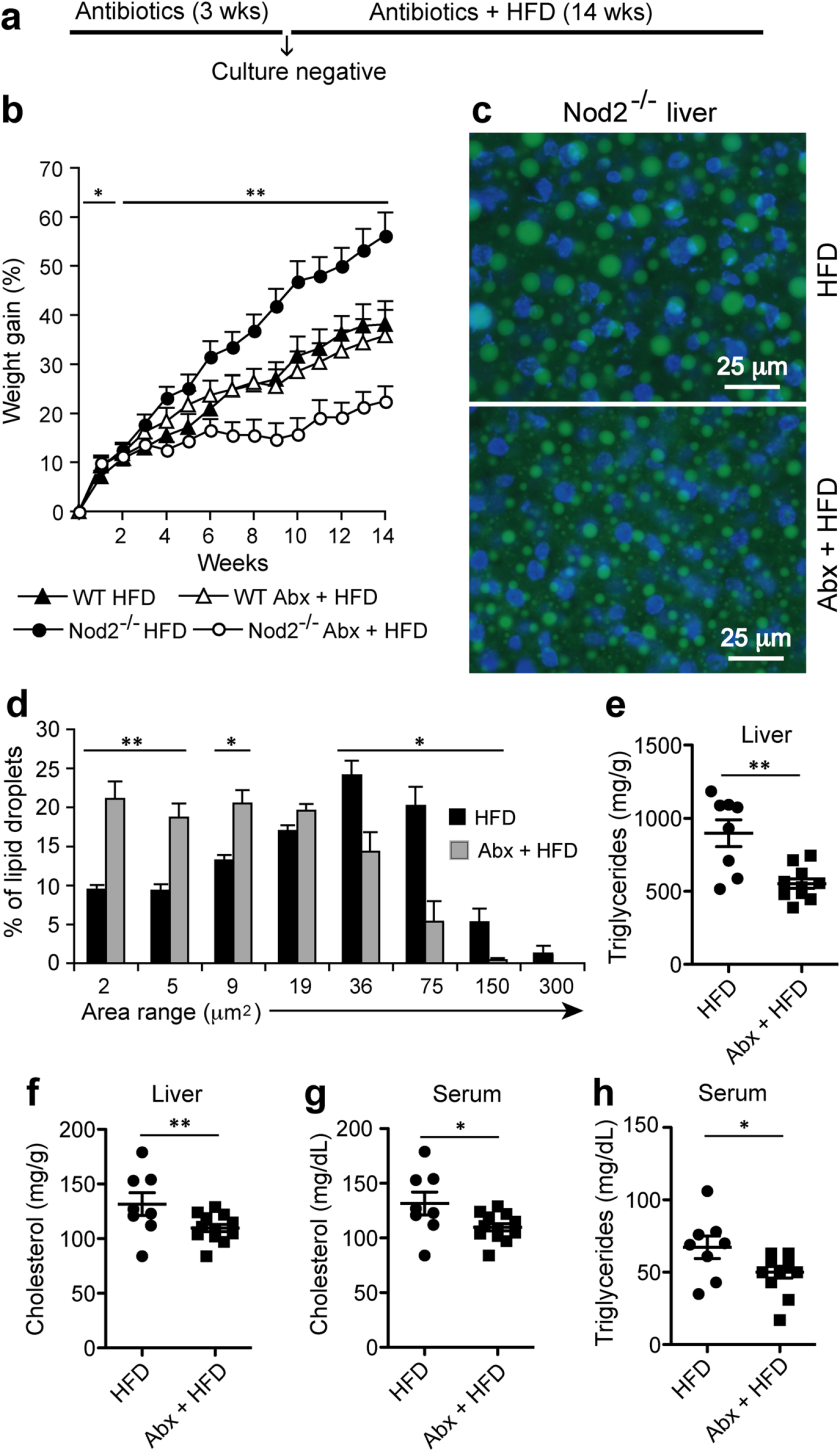
Antibiotic treatment prevents the development of obesity, steatosis, and metabolic dysfunction in *Nod2* ^−/−^ mice on HFD. (**a**) *Nod2* ^−/−^ and WT mice were treated with Abx to deplete their intestinal microbiota. Control mice were not treated with antibiotics. Three weeks after the start of Abx all mice were placed on HFD and monitored for weight gain, histopathology, and metabolites. (**b**) Percent gain in weight compared to week 0 of HFD. (**c**,**d**) After 14 weeks of HFD, mice were analyzed for liver histopathology in BODIPY and DAPI stained sections and (**d**) percent frequency distribution of LD area (µm^2^) for ~4,000 LDs/group. (**e**–**h**) After 14 weeks of HFD, (**e**) liver triglycerides, (**f**) liver cholesterol, (**g**) serum cholesterol, and (**h**) serum triglycerides were measured. (**b**,**d**) Results are means ± SEM, (**C**) representative images, and (**e**–**h**) individual data of 6–10 mice/group. *P ≤ 0.05 and **P ≤ 0.001, *Nod2* ^−/−^ Abx + HFD *versus Nod2* ^−/−^ HFD.

We next determined the role of the gut microbiota in diet-dependent obesity in *Nod2* ^−/−^ mice by depleting gut bacteria with antibiotics. WT and *Nod2* ^−/−^ mice, starting at 4 weeks of age, were given an antibiotic mix (Abx) of ciprofloxacin and metronidazole in their drinking water, which is an established method for depleting gut bacteria^20, 23–25^. After three weeks the animals were placed on HFD and Abx were continued, and body weight was measured each week (Fig. 6a). Control mice were started on HFD at the same age, but never treated with Abx. Our results demonstrate that Abx treatment completely abolished the diet-dependent weight gain in *Nod2* ^−/−^ mice, but did not affect the weight gain in WT mice (Fig. 6b). *Nod2* ^−/−^ Abx HFD mice gained significantly less weight than *Nod2* ^−/−^ HFD mice, and also less than WT HFD mice with and without Abx. There was no difference in weight gained between WT Abx HFD and WT HFD mice. There was no difference in food consumption or water intake between the WT Abx HFD and *Nod2* ^−/−^ Abx HFD, as average food consumed/mouse/day was 1.86 g and 1.89 g, respectively, and the average water intake/mouse/day was 2.9 ml for both genotypes. There is some decrease in water intake between the antibiotic treated versus control mice, however this decrease does not account for the difference in the weight gain observed between WT and *Nod2* ^−/−^ mice, because both genotypes on antibiotics had similar decrease in water intake but only *Nod2* ^−/−^ mice had significantly reduced weight gain.

We next determined whether the antibiotic-induced loss in weight gain was accompanied by decreased lipid accumulation in the liver of *Nod2* ^−/−^ mice using histological and biochemical assays. Our results demonstrate that *Nod2* ^−/−^ mice treated with long-term Abx and maintained on HFD had smaller LDs in hepatocytes compared with *Nod2* ^−/−^ mice not treated with Abx but maintained on HFD (Fig. 6c). The area for LDs ranged from 2 μm^2^ to 300 μm^2^ with the highest percentage of LDs in the 36 to 75 μm^2^ range for the HFD mice and 2 to 19 μm^2^ range for Abx + HFD mice (Fig. 6d). *Nod2* ^−/−^ mice on Abx + HFD had significantly lower levels of liver cholesterol and triglycerides than control *Nod2* ^−/−^ mice on HFD alone (Fig. 6e and f). Furthermore, *Nod2* ^−/−^ mice on Abx + HFD had significantly lower levels of serum cholesterol and triglycerides (Fig. 6g and h), but no significant difference in glucose, compared with control *Nod2* ^−/−^ mice on HFD alone.

Thus, our results demonstrate that the gut microbiota in *Nod2* ^−/−^ HFD mice is required for the development of diet-dependent obesity, steatosis, and hyperlipidemia in *Nod2* ^−/−^ mice, and that long-term treatment with broad-spectrum antibiotics abolishes weight gain and several aspects of metabolic dysfunction.

### Intestinal microbiota from *Nod2*^−/−^ mice on high fat diet increases sensitivity to obesity

**Figure 7:**
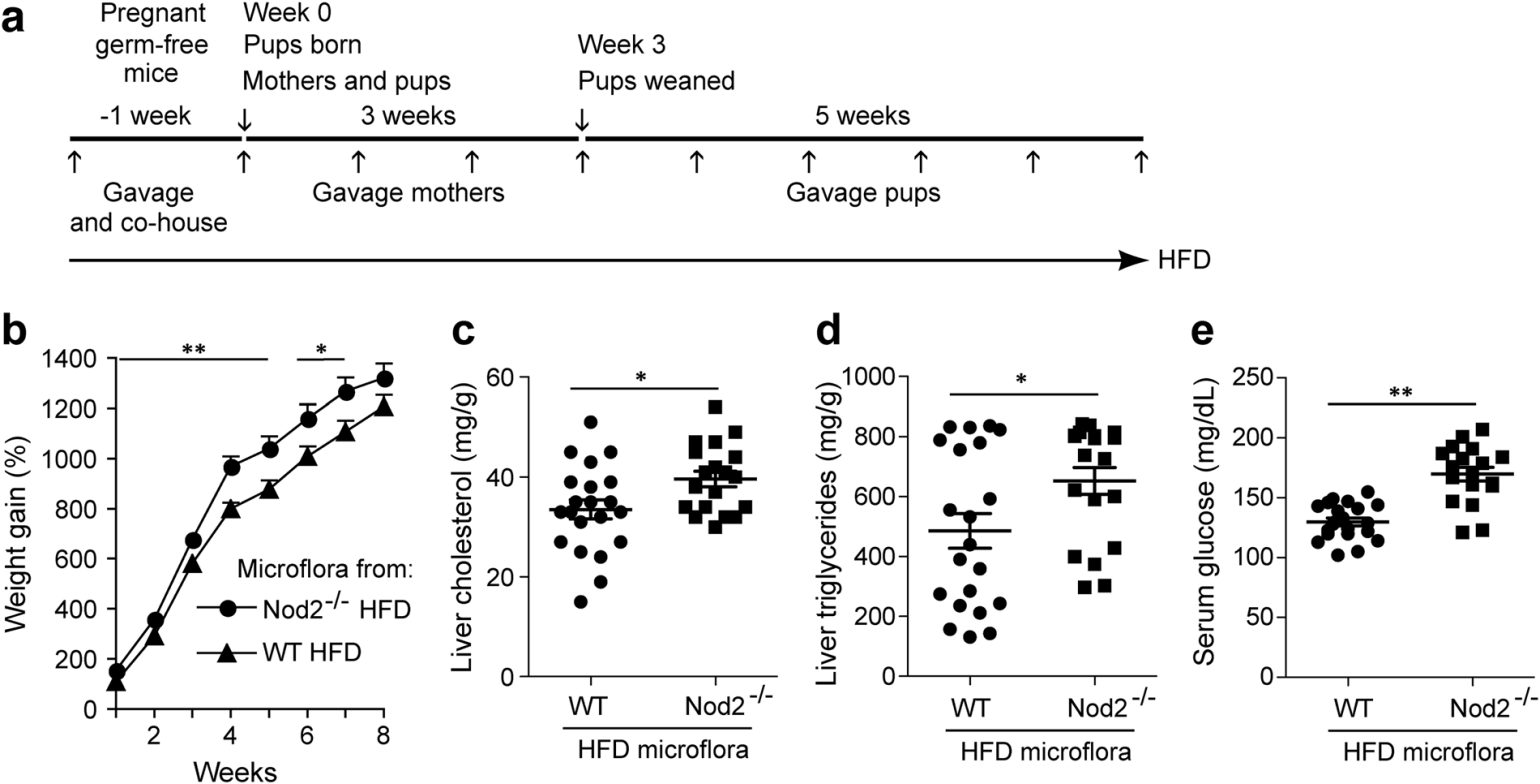
Intestinal microbiota from *Nod2* ^−/−^ mice on HFD increases susceptibility of germ-free mice to HFD-induced obesity and metabolic dysfunction. (**a**) One week before delivery, we colonized germ-free WT Swiss Webster female pregnant mice, kept under sterile conditions, with microbiota from WT or *Nod2* ^−/−^ mice maintained on HFD. Colonization was done by both gavaging with fecal microbiota and by co-housing. Gavaging was done into the stomach with fecal homogenates from WT or *Nod2* ^−/−^ mice maintained on HFD (indicated by ↑). At the same time, the gavaged mice were placed on HFD and also co-housed with *Nod2* ^−/−^ or WT female mice that had been maintained on HFD for ~20 weeks. The co-housed *Nod2* ^−/−^ HFD and WT HFD mice were removed when the pups were born (week 0). Mothers and pups were maintained on HFD for the length of the experiment. (**b**–**e**) Pups were monitored for (**b**) gain in weight every week and after 8 weeks assayed for (**c**) liver cholesterol, (**d**) liver triglycerides and (**e**) serum glucose. (**b**) Results are means ± SEM and (**c**–**e**) individual data of 19–21 mice/group. *P ≤ 0.05 and **P ≤ 0.001, *Nod2* ^−/−^ Abx + HFD *versus Nod2* ^−/−^ HFD.

We next considered whether the altered gut microbiota in *Nod2* ^−/−^ mice on HFD is sufficient for the development of diet dependent obesity. We colonized germ-free pregnant female WT Swiss-Webster mice by co-housing with either *Nod2* ^−/−^ HFD mice or WT HFD mice, and also by weekly gavaging with microbiota from either *Nod2* ^−/−^ HFD mice or WT HFD mice. We placed these mice on HFD after the first gavage (Fig. 7a). We removed the co-housed mice one day after delivery, but continued weekly gavaging and HFD, and monitored the weight gain of pups for 8 weeks. Germ-free pups colonized with microbiota from *Nod2* ^−/−^ HFD mice were more susceptible to HFD-dependent weight gain than pups colonized with microbiota from WT HFD mice, as manifested by significantly higher weight gain (Fig. 7b). Furthermore, pups colonized with *Nod2* ^−/−^ HFD microbiota were also more susceptible to HFD-induced metabolic dysfunction, as they had significantly higher liver cholesterol and triglycerides, and higher serum glucose than pups colonized with microbiota from WT HFD mice (Fig. 7c–e), but there was no difference in serum cholesterol or triglycerides between the 2 groups of mice. In conclusion, microbiota from *Nod2* ^−/−^ HFD mice can transfer to WT outbred germ-free mice enhanced susceptibility to HFD-dependent weight gain and metabolic dysfunction.

## Discussion

Multiple factors, including host genetics, microbiota, and the environment regulate sensitivity to obesity and metabolic disease and development of their clinical manifestations. However, the specific components that promote or protect from these diseases are poorly understood. There is increasing evidence for cross talk between innate immunity and metabolism and for their combined role in the development of obesity and associated diseases^6, 26–28^. However there is no previous evidence for the role of the innate immunity gene *Nod2* in regulating sensitivity to obesity. Here we have discovered that *Nod2* and *Nod2*-dependent microbiota protect BALB/c mice from HFD-induced pathology.

We show that *Nod2* ^−/−^ BALB/c mice on HFD become obese and exhibit many key manifestations of metabolic disease including hypercholesterolemia, hypertriglyceridemia, hyperglycemia, and hyperinsulinemia, increased adiposity, and steatosis. The effect of *Nod2* deletion on HFD-induced obesity is strain-dependent, as *Nod2* ^−/−^ and WT C57BL/6 mice on HFD gain similar weight, but *Nod2* ^−/−^ C57BL/6 mice exhibit more severe insulin resistance^21^ than *Nod2* ^−/−^ BALB/c mice. Thus, *Nod2* differentially regulates the response of BALB/c and C57BL/6 mice to HFD, which emphasizes the importance of the host’s entire genetic background in regulation of obesity and metabolic syndrome and reflects the variability in the development of disease in humans.

Metabolic inflammation plays a key role in the development of obesity and metabolic diseases in humans and in animal models of obesity^5, 6^. Our results also demonstrate that HFD-dependent obesity in *Nod2* ^−/−^ mice correlates with increased expression of a large number of genes in adipose tissue that participate in immune responses. They include genes for chemokines and cell surface molecules involved in the recruitment and activation of T cells, monocytes, dendritic cells and eosinophils, and for enzymes in eicosanoid metabolism. In addition, *Nod2* ^−/−^ HFD mice have increased infiltration of F4/80^+^ macrophages and enhanced levels of the transcripts of *Adgre1* (gene for F4/80) and *CD68*, also a marker for monocytes/macrophages^29^, in the adipose tissue. Similar to our results, increased inflammatory markers were also observed in *Nod2* ^−/−^ C57BL/6 mice on HFD^21^, which suggests that inflammation likely plays an important role in the development of metabolic dysfunction in both strains of mice.

Development of obesity is usually accompanied by dysfunction in lipid and/or carbohydrate metabolism, and our results also demonstrate that HFD-dependent obesity in *Nod2* ^−/−^ mice correlates with changes in the expression of many genes involved in intermediary metabolism, primarily lipid metabolism. Several of these differentially expressed genes and their products have an established role in the development of obesity or are associated with metabolic diseases^30–35^. Leptin is an adipokine that regulates appetite and energy balance, and in obese individuals there is decreased sensitivity to leptin, which may result in increased appetite^36^. *Nod2* ^−/−^ mice on HFD have elevated levels of serum leptin, enhanced transcription of leptin (*Lep*) in the adipose tissue, and decreased expression of the leptin receptor in the liver (*Lepr*). These results suggest that *Nod2* ^−/−^ mice on HFD develop resistance to leptin, however there was no difference in the amount of food consumed between WT and *Nod2* ^−/−^ mice on HFD. Genes known to participate in multiple other biological pathways, including DNA structure, gene expression, cell cycle, development, neural processes, transport, cytoskeleton, and extracellular matrix were also differentially expressed between *Nod2* ^−/−^ HFD mice compared with WT HFD mice, which suggest the development of a complex pathology.

Obesity is often associated with non-alcoholic fatty liver disease, and *Nod2* ^−/−^ mice on HFD also exhibit similar manifestations, including enlarged fatty liver, elevated levels of cholesterol and triglycerides in the liver, and large LDs in the hepatocytes. LDs are composed of a lipid core surrounded by a phospholipid monolayer and are coated with proteins that regulate LD physiology, including enzymes in lipid metabolism and members of the perilipin family, which regulate lipid metabolism and trafficking^37, 38^. The development of LDs is regulated by multiple factors, including host genetics, composition of the intestinal microbiota, and diet^37–40^. Using proteomics we identified many proteins in the LD enriched fraction with significantly changed abundance in *Nod2* ^−/−^ HFD mice compared with WT HFD mice. All identified proteins have known functions in lipid metabolism and/or vesicle trafficking and several of these proteins, for example Abhd5, Pnpla3, and COPI, have an established role or are associated with obesity and /or steatosis^37, 38, 41–45^. In addition, many genes involved in lipid synthesis, degradation, and storage were differentially regulated in the liver of *Nod2* ^−/−^ mice on HFD compared with WT mice on HFD. These results, thus, provide clues for the molecular mechanism responsible for the development of steatosis in *Nod2* ^−/−^ HFD mice. Moreover, the altered microbiota in *Nod2* ^−/−^ HFD mice also contributes to increased lipid accumulation in hepatocytes and may play a role in the formation of large LDs.

Obese mice and humans often show increased abundance of *Firmicutes* and *Proteobacteria*, but decreased abundance of *Bacteroidetes* ^3, 46, 47^. Accordingly, our results also show higher abundance of six *Firmicutes* species and one *Proteobacteria* species in obese *Nod2* ^−/−^ HFD mice compared with non-obese WT HFD mice. The abundance of several of these species is also increased in obese humans^48–51^. Our results comparing the abundance of *Bacteroidetes* in obese and non-obese mice were mixed: three *Bacteroidetes* species were increased and two were decreased in *Nod2* ^−/−^ HFD mice compared with WT HFD mice, consistent with many exceptions to the general rule of decreased *Bacteroidetes* in obesity, e.g., increased abundance of *Bacteroides* was found in some obese and obese-prone populations^52, 53^. Also, consistent with the association of inflammation with obesity, the abundance of two members of *Deferribacteres* were increased in obese *Nod2* ^−/−^ HFD mice compared with all other non-obese groups of mice, as *Deferribacteres* are early markers of inflammation^54^. Our results concur with previous data showing dysbiosis in *Nod2* ^−/−^ C57BL/6 mice on HFD^21^, but the specific differences were not the same as those seen in *Nod2* ^−/−^ BALB/c mice on HFD.

There is increasing evidence indicating a role for the gut microbiota in the development of obesity in humans and in animal models of obesity^2, 3^. Microbiota with increased capacity for harvesting energy, referred to as “obese microbiota”, has been identified in obese individuals, and furthermore, the obese microbiota from humans can transfer the obesity phenotype to mice^55, 56^. Our results showing abrogation of obesity and metabolic changes in *Nod2* ^−/−^ HFD mice by antibiotics and transfer of increased sensitivity to obesity in germ-free mice by microbiota from *Nod2* ^−/−^ HFD mice suggest that *Nod2* may protect mice from harboring such “obese microbiota”. This notion is further supported by a decreased total energy content in the stools of *Nod2* ^−/−^ HFD mice compared with WT HFD mice, suggesting greater extraction of energy from food in *Nod2* ^−/−^ HFD mice, leading to obesity and metabolic disease. These results indicate that there are many similarities in the mechanisms responsible for microbiota-induced obesity in humans and mice, although the specific bacteria present in humans and mice may differ.

In summary, we have discovered that deletion of *Nod2* abolishes the resistance of BALB/c mice to HFD-induced obesity and metabolic dysfunction. The development of obesity and metabolic dysfunction in *Nod2* ^−/−^ mice on HFD has many similarities to the development of HFD-induced pathology in humans, which makes *Nod2* ^−/−^ BALB/c mice a relevant model for the study of HFD-induced obesity and associated diseases.

## Methods

### Mice

*Nod2* ^−/−^ mice on BALB/c background were described previously and deletion of the *Nod2* genes was confirmed by PCR analysis of genomic DNA^19, 20^. The original founder WT BALB/c mice were obtained from Harlan-Sprague-Dawly. WT germ-free mice (Swiss Webster female and male) were obtained from Taconic Farms (Hudson, NY). All WT and *Nod2* ^−/−^ mice on BALB/c background were bred and kept under conventional pathogen-free conditions in the same room in our facility to minimize the influence of differences in the environment. For each experiment, mice from several different cages and breeder pairs were used. To avoid changes in microbiome that could accumulate over extended period of time, we backcross our mutant mice to WT females once every other year and re-derive our homozygous knockout breeding pairs. The latter strategy also minimized genetic drift in the population. Some investigators use knockout and WT littermates from heterozygous breeding pairs to minimize parent-to-parent and cage-to-cage variations. We do not use this strategy for two reasons: first, this strategy may skew the results to the particular microbiota present only in this breeding pair. Second and most importantly, we noticed that the effect of innate immunity genes on the composition of microbiota is not instantaneous, but takes time, and stabilization of microbiota characteristic of a given knockout may take more than one generation^20^. The BALB/c background of knockout mice and their negative status for all common viral and bacterial pathogens and parasites were confirmed as previously described^19^. The Indiana University School of Medicine-Northwest Institutional Animal Care and Use Committee approved all experiments with mice. All methods were performed in accordance with the relevant guidelines and regulations.

### Obesity model in conventional mice

WT and *Nod2* ^−/−^ female mice in BALB/c background, matched for weight and age (6–7 weeks old) were randomly divided into two groups and started on HFD (fat 60 kcal%, D12492) or LFD (fat 10 kcal%, D12450B) from Research Diets and maintained on the special diets until the end of the experiment (14 or 30 weeks). The total calories/g of HFD and LFD were the same. Mice were weighed every week and the amount of food consumed each week was measured to confirm that the differences in obesity are not due to differences in food intake. One to two weeks before sacrifice, stool samples were collected for microbiota analysis, bomb calorimetry and for microbiota transplant experiments, fasting glucose was assayed, and glucose tolerance and insulin resistance assays performed. At the time of sacrifice, blood was collected for biochemical assays; visceral adipose tissue and liver were weighed and subcutaneous adipose tissue was measured with a micrometer. Visceral adipose tissue and liver were processed for histology, RNA, and flow cytometry. Lipid droplets were purified from the liver and proteins identified by mass spectrometry. We focused on adipose tissue and liver, because they are the major organs involved in intermediary metabolism.

### Obesity in antibiotic-treated mice

We depleted the microbiota in WT and *Nod2* ^−/−^ mice using an established antibiotic treatment^20, 23–25^. Mice were matched for weight and age and randomly divided into two groups. One group of 4 weeks old mice was started on an antibiotic mix (Abx) of ciprofloxacin (100 µg/ml) and metronidazole (250 µg/ml) in their drinking water. After three weeks of Abx treatment anaerobic stool cultures were negative (<10^3^ bacteria/g of feces, freshly collected in reducing buffer), and mice were placed on HFD and Abx was continued, and body weight and food consumed were measured each week. Control mice were started on HFD at the same age, but not treated with Abx. At 14 weeks after the start of HFD, mice were sacrificed, blood was collected for biochemical assays and liver was processed for histology and lipid assays.

### Obesity in microbiota transplanted germ-free mice

One week before delivery, we colonized germ-free WT Swiss Webster female pregnant mice, kept under sterile conditions, with microbiota from WT or *Nod2* ^−/−^ mice maintained on HFD. Colonization was done by both gavaging with fecal microbiota and by co-housing. Gavaging was done into the stomach with 10 mg of fecal homogenates (containing 5 × 10^9^ bacteria in 0.2 ml) prepared from freshly defecated stool samples, collected from WT or *Nod2* ^−/−^ mice maintained on HFD for 29–30 weeks, and immediately placed in reduced anaerobic medium, homogenized, and stored frozen at −80 °C, as previously described^20, 25^. At the same time, the gavaged mice were placed on HFD and also co-housing with *Nod2* ^−/−^ or WT female mice that had been maintained on HFD for 20 weeks was started and continued until one day after delivery. The mothers were gavaged weekly and maintained on HFD until weaning of the pups at 3 weeks of age. Pups were gavaged at weaning and once a week thereafter, and continued on HFD. Body weight was measured every week and blood was collected at the time of sacrifice. We used this strategy to ensure successful colonization with microbiota, since we did not know whether gavaging or co-housing would be more effective in transferring obesity-promoting microbiota. We colonized pregnant mice to ensure that the pups will be born colonized with a specific gut microbiota. The presence of microbiota at birth is essential for the normal development of host metabolism and immune systems and alterations in the gut bacteria at birth increases susceptibility to diet-induced obesity^57^.

### Histopathology of liver and adipose tissue

Liver and adipose tissue were fixed in 10% buffered formalin, embedded in paraffin, sectioned, and stained with hematoxylin and eosin. Because lipid is lost with paraffin embedding, we also analyzed frozen liver sections stained with BODIPY 493/503 (Molecular Probes) and DAPI (Vector Laboratories). The liver sections were fixed in 4% paraformaldehyde/PBS for 10 min at room temperature, rinsed with PBS, and stained with 1 µg/ml of BODIPY 493/503 in PBS for 5 min at room temperature. The sections were rinsed with PBS and then covered with Vectashield mounting medium containing DAPI. The area for lipid droplets and adipocytes was measured using ImageJ and Adiposoft^58^, respectively.

### Biochemical analyses

Mice were fasted for 5 h and blood was collected. Serum cholesterol and triglycerides were quantified using fluorometric kits from BioVision Inc. Serum insulin and leptin were analyzed using ELISA kits from Linco Research Inc. Blood glucose was measured using the CVS/pharmacy Advanced Glucose Meter and test strips. Cholesterol and triglycerides were also quantified in liver homogenates (BioVision Inc).

### Glucose tolerance test and estimation of insulin sensitivity

WT and *Nod2* ^−/−^ mice maintained on HFD for 29 weeks were fasted for 5 h and baseline glucose measured using blood collected from the tail vein. For the glucose tolerance test, mice were injected intraperitoneally with 2 g glucose/kg body weight in sterile PBS and blood glucose measured at 40, 80, and 120 min after injection. For the insulin sensitivity test, mice were injected intraperitoneally with 0.4 U/kg body weight of Humulin-R insulin (HealthWarehouse) in sterile PBS and blood glucose measured at 15, 30, 60, 90, and 120 min after injection.

### Bomb calorimetry

To identify changes in energy extracted from the food between *Nod2* ^−/−^ and WT mice on HFD, fresh fecal samples were collected from individual mice, weighed, and frozen at −80 °C until processing. Samples were dried at 58 °C for 24 h, dried pellets were ground and energy content was measured using a Parr Oxygen Bomb Calorimeter and total caloric value computed as per the manufacturers instructions.

### Lipid droplet enrichment and protein analysis

#### Lipid droplet enrichment

We enriched LDs using a modified procedure^59^. Freshly dissected livers were homogenized on ice with a glass Dounce homogenizer containing ice-cold buffer A (20 mM tricine and 250 mM sucrose, pH 7.8) with 0.2 mM PMSF and protease inhibitor cocktail. The homogenate was centrifuged at 100 g for 10 min at 4 °C. The supernatant was further disrupted for 15 min at 500 psi in a nitrogen bomb chamber and centrifuged at 3,000 g for 10 min at 4 °C to remove nuclei, cell debris, and unbroken cells. The post-nuclear supernatant (PNS) was transferred into a 15 ml tube and 3 ml of buffer B (20 mM HEPES, 100 mM KCl and 2 mM MgCl_2_, pH 7.4) was loaded on top and centrifuged at 2,000 g for 30 min at 4 °C. LDs were collected from the top band and washed several times with 200 μl buffer B and centrifuged at 20,000 g for 5 min at 4 °C. The samples were mixed with chloroform/acetone (0.25:0.75, v/v), kept at −80 °C overnight, and proteins and lipids were separated by centrifugation at 20,000 g for 10 min at 4 °C. The protein pellet was air-dried, dissolved in 8 M Urea and analyzed by mass spectrometry.

#### Proteomic analysis by mass spectrometry

Proteins in the LD enriched fraction were identified by mass spectrometry at the Laboratory for Biological Mass Spectrometry, Indiana University Bloomington. Proteins were digested with trypsin and purified peptides were run on an Eksigent Nano 2D HPLC coupled to a LTQ Velos Pro mass spectrometer (Thermo Fisher Scientific, Waltham MA). The resulting data were searched in Protein Prospector against the *Homo sapiens* or *Mus musculus* proteome in the SwissProt database. Spectral counting was used for protein quantification. Normalized data for comparison were obtained by dividing spectral count of a single protein by the sum of spectral counts in the same sample. The fold change was computed for *Nod2* ^−/−^ HFD with respect to WT HFD and genes with a fold change of ≥1.5 or ≤0.6 and *P* ≤ 0.05 were considered as significantly different between the 2 groups of mice.

### RNA transcriptomics

For identification of genes that were differentially expressed between *Nod2* ^−/−^ and WT mice on HFD, we analyzed the total RNA population in the liver and adipose tissue by RNA sequencing. RNA was isolated from the liver and adipose tissue of individual WT and *Nod2* ^−/−^ mice maintained on HFD for 30 weeks using the TRIZOL method (InVitrogen), followed by purification on RNeasy spin columns using Qiagen RNeasy Mini Kit^60^. RNA sequencing and initial analysis were performed at the Center for Genomics and Bioinformatics, Indiana University Bloomington. 98% of all reads (~311 million reads) passed a quality filter of q20 requiring a minimum length of 50 bases after adapter and quality trimming, 94.7% of these mapped to the mouse genome assembly GRCm38.75 using tophat ver.2.0.10 and 95.5% of these mapped uniquely. The reads were annotated using the software DAVID^61^ and differential expression with statistical analysis was calculated using the software DESeq2^62^. The fold change was computed for *Nod2* ^−/−^ HFD with respect to WT HFD and genes that had a fold change of ≥2 or ≤0.5 and *P* ≤ 0.05 and with a 5% FDR were considered as significantly different between the 2 groups.

### Isolation of stromal/vascular cells from adipose tissue and flow cytometry

Stromal/vascular fraction from adipose tissue, which contains immune cells, was isolated using a modified procedure^63^. Visceral adipose tissue was isolated, weighed, and digested with 1 mg/ml collagenase D and 0.15 μg/ml DNAse I in RPMI-5FCS (RPMI-1640 supplemented with 5% FCS) for 40 min in a shaking 37 °C water bath and samples were centrifuged at 1,000 rpm for 10 min at room temperature; at this speed adipocytes stay on the top and the stromal/vascular (SV) cells form a pellet. The supernatant with the adipocytes was carefully removed and discarded and the pellet was resuspended in RPMI-5FCS and filtered through a 100 μm filter to remove any remaining adipocytes. SV cells were stained with CD45-Vioblue and F4/80-APC (Biolegend) for 20 min, analyzed by flow cytometry using MACSQuant (Miltenyi) cytometer and F4/80 positive cells were measured within the CD45 gate.

### Gut microbiome analysis

#### Stool collection and DNA extraction

We collected freshly defecated stools from individual female WT and *Nod2* ^−/−^ mice (200 mg/mouse, 6 mice/strain) maintained for 28–30 weeks on HFD or LFD and isolated DNA using the MoBio Fecal DNA isolation kit^25^. For each genotype, mice originated from three different litters from different parents and 2 mice per litter), weaned into separate cages, and all cages were kept in the same room in our animal facility. This strategy allows stabilization of microbiota and minimizes the variability observed between different litters due to different parents and different cages and rooms^20, 25, 60^.

#### Pyrosequencing of 16S rRNA genes and microbiome analysis

Genetic phylotyping (community profiling) using pyrosequencing of the variable regions of bacterial 16 S ribosomal RNA (rRNA) genes with 16 S universal Eubacterial primers 530 F (5′-GTG CCA GCMGCN GCG G) and 1100 R (5′-GGG TTN CGN TCG TTG) was performed using Roche 454 technology and bTEFAP® pipeline at MR DNA Molecular Research LP^25, 64^. A single-step 30 cycle PCR using HotStarTaq Plus Master Mix Kit (Qiagen) was used under the following conditions: 94 °C for 3 min, followed by 28 cycles of 94 °C for 30 sec, 53 °C for 40 sec, and 72 °C for 1 min, and elongation step at 72 °C for 5 min. Following PCR, all amplicon products from different samples were mixed in equal concentrations and purified using Agencourt Ampure beads (Agencourt Bioscience Corporation). Samples were sequenced using Roche 454 FLX titanium instrument and reagents by following manufacturer’s guidelines. Sequencing data were processed using MR DNA Molecular Research LP analysis pipeline (www.mrdnalab.com), including depletion of barcodes and primers, removal of short sequences (<200 bp), sequences with ambiguous base calls, and sequences with homopolymer runs exceeding 6 bp, de-noising, and removal of chimeras^65^. Operational taxonomic units (OTUs) were then defined after removal of singleton sequences and clustering, and taxonomically classified using BLASTn against a curated GreenGenes database and assigned to species at >97% identity to the reference sequence (<3% divergence), to genus at 95%–97% identity, family at 90%–95% identity, order at 85%–90% identity, class at 80%-85% identity, phylum at 77%-80% identity, and unclassified at <77% identity^66^. We compared the sequences and analyzed the changes in bacterial ecology using Quantitative Insights Into Microbial Ecology (QIIME; http://qiime.sourceforge.net), Bayesian, PyNAST, and UniFrac^67–69^. The Shannon diversity index H = –Σ*p*_*i*_ ln(*p*_*i*_) and Shannon equitability index E_H_ = H/ln(S), where *p*_*i*_ is the proportion of the *i*th taxonomic unit (OTU or species) and S is the total number of taxonomic units, were calculated using Microsoft Excel. The average total number of reads per mouse was 6978, with no significant differences between the numbers of reads/mouse between WT and *Nod2* ^−/−^ and HFD and LFD mice, and detected bacteria with a frequency of >0.001%.

### Statistical analysis

The significance of differences between the numbers of recovered bacterial species and OTUs was determined by the *z*-test using Microsoft Excel. The significance of differences between all other quantitative results presented as means ± SEM were determined by the Student’s *t*-test using Microsoft Excel or by ANOVA using Prism. The *N* and *P* values are indicated in the Figures and Tables; *P* ≤ 0.05 was considered significant. The heatmaps were generated using Java TreeView and represent mean fold changes in *Nod2* ^−/−^ HFD mice relative to WT HFD mice, after converting <1 ratios to negative fold difference using the formula: (−1)/ ratio.

## Electronic supplementary material

Supplementary Table 1

Supplementary Table 2

Supplementary Figures
