# Author Correction: *Nod2* and *Nod2*-regulated microbiota protect BALB/c mice from diet-induced obesity and metabolic dysfunction

**DOI:** 10.1038/s41598-018-24594-7

**Published:** 2018-04-16

**Authors:** Ivan Rodriguez-Nunez, Tiffany Caluag, Kori Kirby, Charles N. Rudick, Roman Dziarski, Dipika Gupta

**Affiliations:** Indiana University School of Medicine–Northwest, Gary, IN 46408 USA

Correction to: *Scientific Reports* 10.1038/s41598-017-00484-2, published online 03 April 2017

In Figures 2e, 6e and 7d, the Y axis scale is incorrect. The correct Figures appear below as Figures 1, 2 and 3.

**Figure 1:**
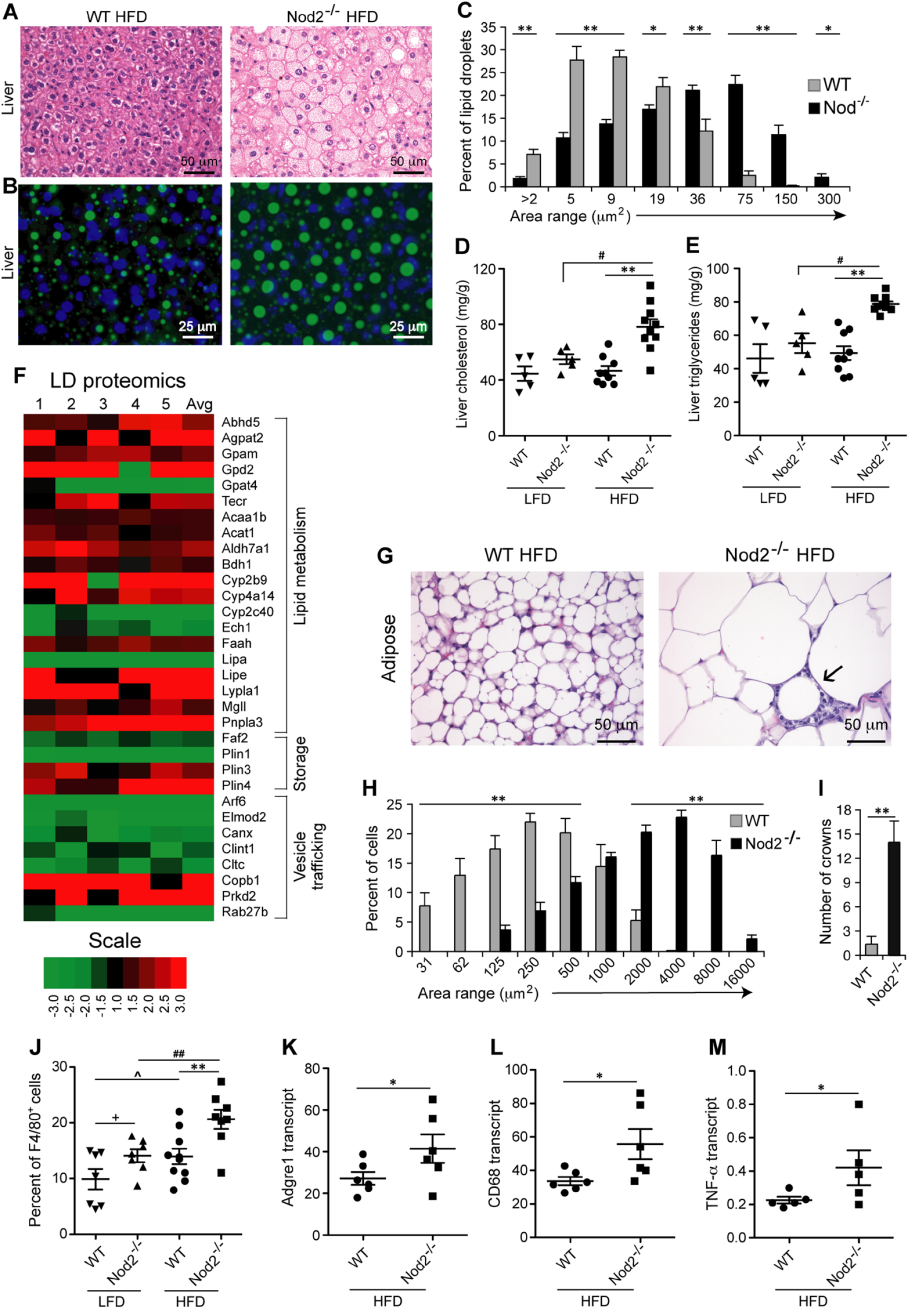
*Nod2*^−/−^ mice on HFD develop steatosis and have large stressed adipocytes with increased infiltration of macrophages. (**A**,**B**) Liver sections from *Nod2*^−/−^ and WT mice stained with (**A**) H&E or (**B**) BODIPY and DAPI. (**C**) Percent frequency distribution of the area (μm^2^) of ~5,000 LDs/group. *(***D**,**E**) Quantification of liver cholesterol and triglycerides. (**F**) Heatmap representation of the fold increase (red) or decrease (green) in the abundance of proteins present in LD-enriched fraction from liver. The fold ratio for individual *Nod2*^−/−^ HFD mice to the average of WT HFD mice were computed (lanes 1 to 5) with the average of the fold for all *Nod2*^−/−^ HFD mice. (**G**) Adipose tissue H&E sections and a dying cell (→) is indicated. (**H**) Percent frequency distribution the area of ~8,000 adipocytes/group. (**I**) Average numbers of dying adipocytes (crowns) per 3,000 cells in H&E stained sections. (**J**) Quantification of CD45+F4/80+ macrophage in adipose tissue. (**K**–**M**) Transcript levels for *Adgre1*, *Cd68*, and *Tnfα* in adipose tissue. Images are representative and results are (**C**,**H**,**I**) means ± SEM of 6 mice/group and (**D**,**E**,**J**–**M**) for individual mice with *N* = 5–10 mice/group. *P ≤ 0.05, **P ≤ 0.001, *Nod2*^−/−^ HFD *versus* WT HFD; ^#^*P* ≤ 0.05, ^##^*P* ≤ 0.001, *Nod2*^−/−^ HFD *versus Nod2*^−/−^ LFD; ^*P* ≤ 0.05, WT HFD *versus* WT LFD; and ^+^*P* ≤ 0.05, *Nod2*^−/−^ LFD *versus* WT LFD.

**Figure 2:**
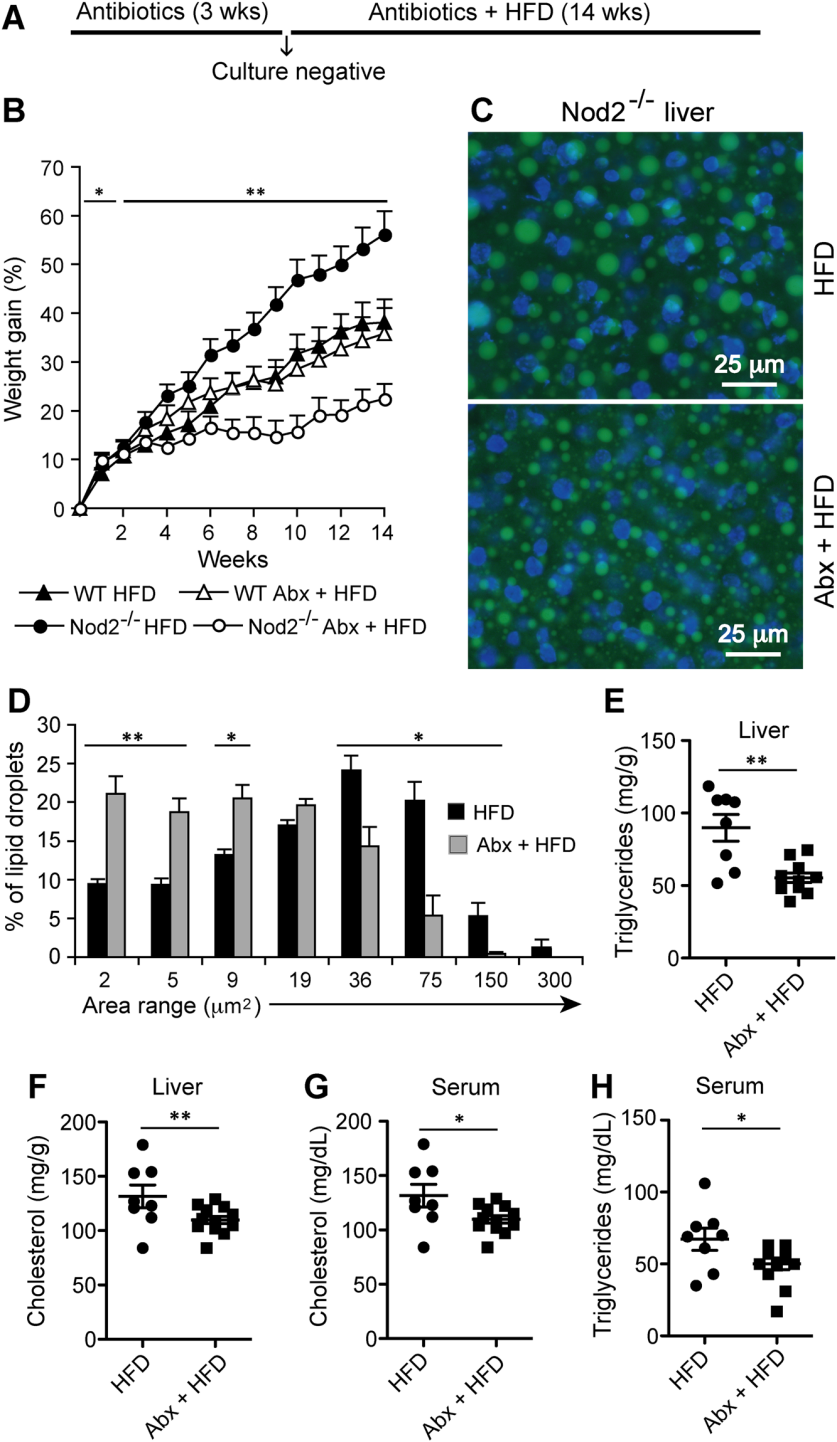
Antibiotic treatment prevents the development of obesity, steatosis, and metabolic dysfunction in *Nod2*^−/−^ mice on HFD. (**A**) *Nod2*^−/−^ and WT mice were treated with Abx to deplete their intestinal microbiota. Control mice were not treated with antibiotics. Three weeks after the start of Abx all mice were placed on HFD and monitored for weight gain, histopathology, and metabolites. (**B**) Percent gain in weight compared to week 0 of HFD. (**C**,**D**) After 14 weeks of HFD, mice were analyzed for liver histopathology in BODIPY and DAPI stained sections and (**D**) percent frequency distribution of LD area (μm^2^) for ~4,000 LDs/group. (**E**–**H**) After 14 weeks of HFD, (**E**) liver triglycerides, (**F**) liver cholesterol, (**G**) serum cholesterol, and (**H**) serum triglycerides were measured. (**B**,**D**) Results are means ± SEM, (**C**) representative images, and (**E**–**H**) individual data of 6–10 mice/group. *P ≤ 0.05 and **P ≤ 0.001, *Nod2*^−/−^ Abx + HFD *versus Nod2*^−/−^ HFD.

**Figure 3:**
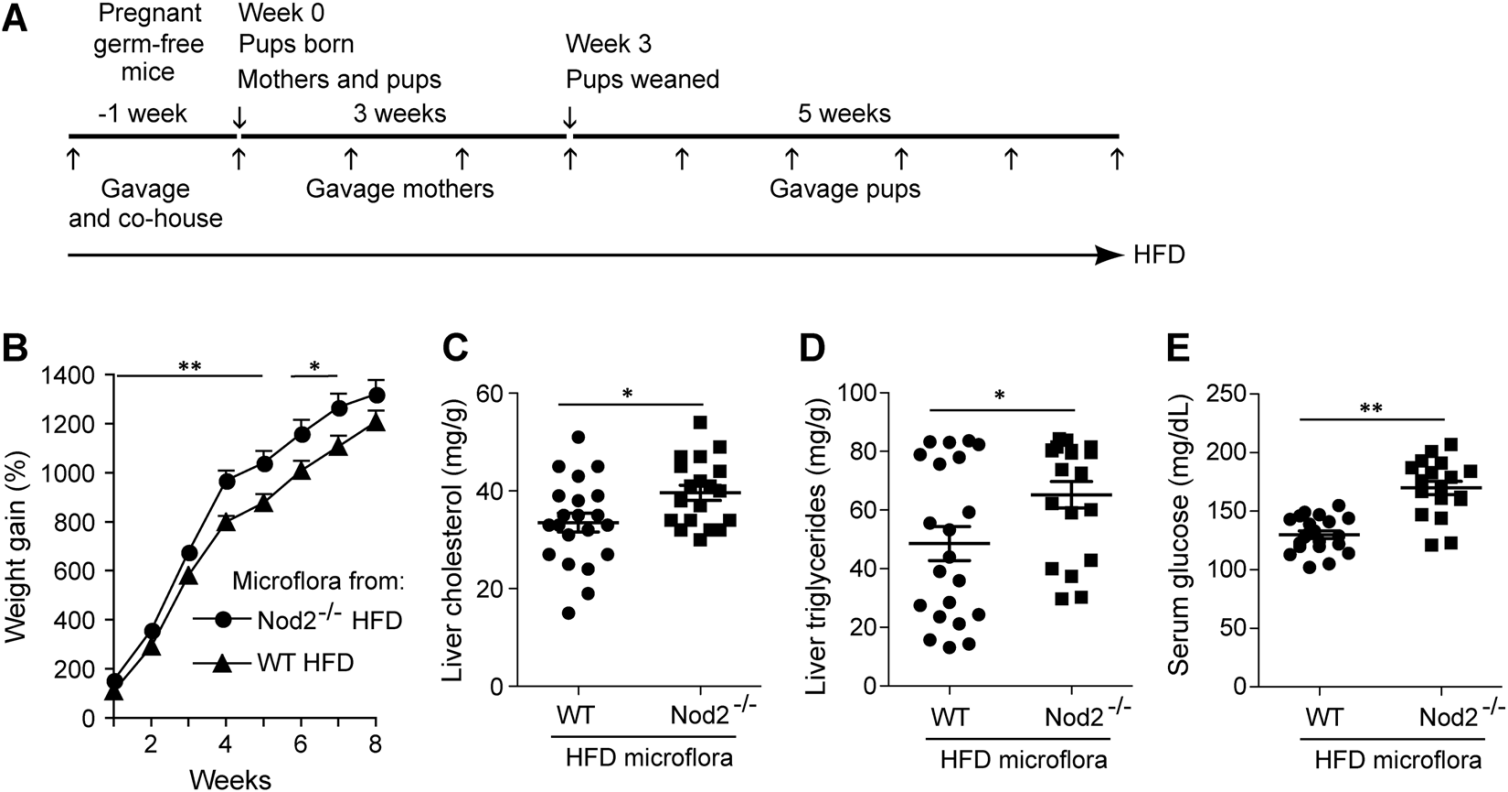
Intestinal microbiota from *Nod2*^−/−^ mice on HFD increases susceptibility of germ-free mice to HFD-induced obesity and metabolic dysfunction. (**A**) One week before delivery, we colonized germ-free WT Swiss Webster female pregnant mice, kept under sterile conditions, with microbiota from WT or *Nod2*^−/−^ mice maintained on HFD. Colonization was done by both gavaging with fecal microbiota and by co-housing. Gavaging was done into the stomach with fecal homogenates from WT or *Nod2*^−/−^ mice maintained on HFD (indicated by ↑). At the same time, the gavaged mice were placed on HFD and also co-housed with *Nod2*^−/−^ or WT female mice that had been maintained on HFD for ~20 weeks. The co-housed *Nod2*^−/−^ HFD and WT HFD mice were removed when the pups were born (week 0). Mothers and pups were maintained on HFD for the length of the experiment. (**B**–**E**) Pups were monitored for (**B**) gain in weight every week and after 8 weeks assayed for (**C**) liver cholesterol, (**D**) liver triglycerides and (**E**) serum glucose. (**B**) Results are means ± SEM and (**C**–**E**) individual data of 19–21 mice/group. *P ≤ 0.05 and **P ≤ 0.001, *Nod2*^−/−^ Abx + HFD *versus Nod2*^−/−^ HFD.

