# Supplementary material for: Nod2 and Nod2-regulated microbiota protect BALB/c mice from diet-induced obesity and metabolic dysfunction
Source: Sci Rep. 2017 Apr 3;7:548. doi: 10.1038/s41598-017-00484-2 (PMC5428441; doi:10.1038/s41598-017-00484-2)
Supplement: Supplementary file 3 — Supplementary Figures [file 41598_2017_484_MOESM3_ESM.doc]

***Nod2* and *Nod2*-regulated microbiota protect BALB/c mice from diet-induced obesity and metabolic dysfunction**

Ivan Rodriguez-Nunez, Tiffany Caluag, Kori Kirby, Charles N. Rudick, Roman Dziarski, and Dipika Gupta

**Supplementary Figures**


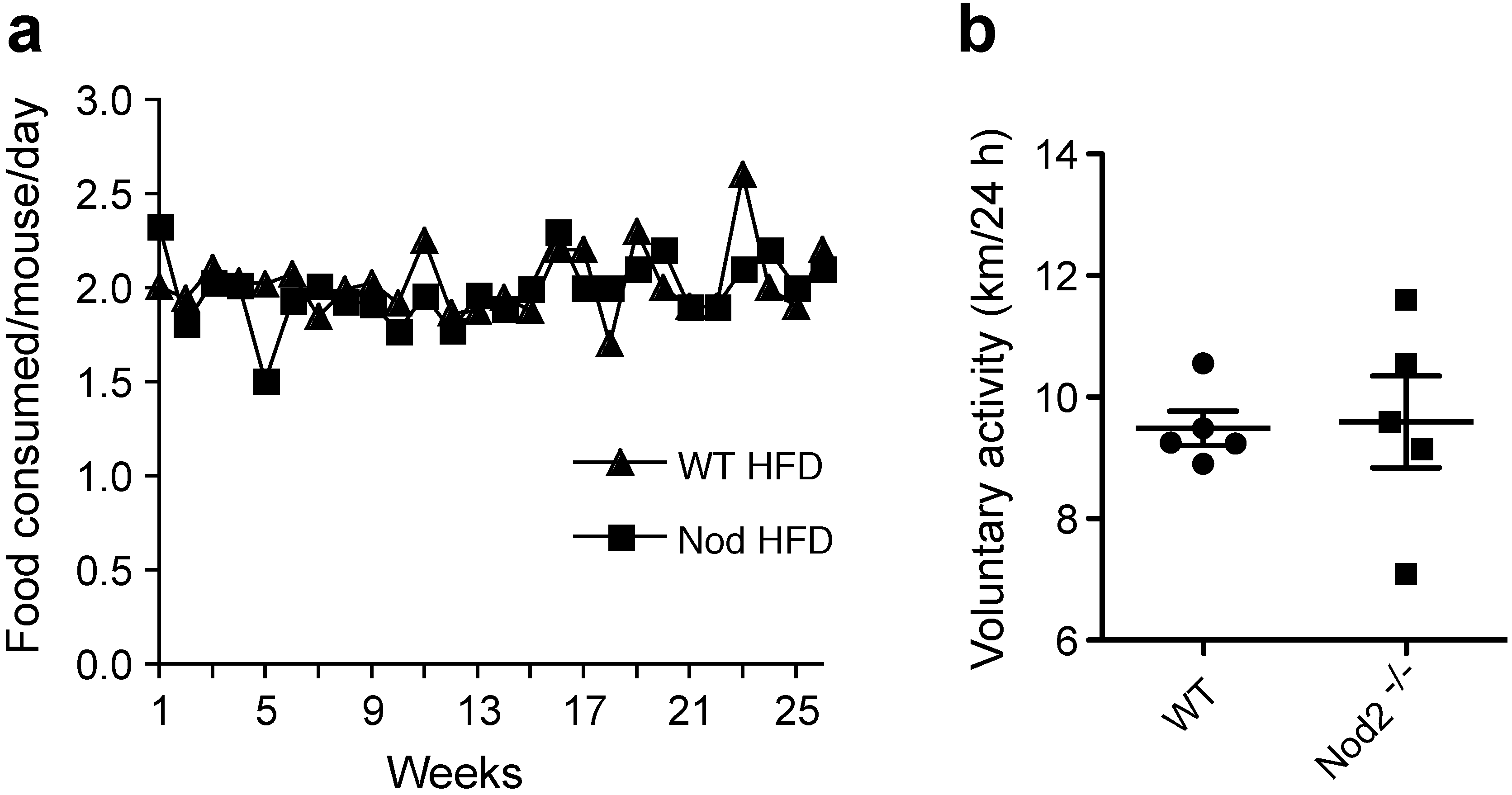


**Supplementary Figure S1. *Nod2*^-/-^ and WT mice on HFD show no difference in the amount of food consumed and *Nod2*^-/-^ and WT mice on regular chow show no difference in voluntary activity.** (a) The amount of food consumed by WT and *Nod2*^-/-^ mice on HFD per cage was measured each week for 30 weeks and the amount of food consumed per mouse is shown. Results are means of 4 cages for each group, and total number of mice, *N*=20-24. (b) *Nod2*^-/-^ and WT mice on regular chow have no difference in voluntary wheel activity.


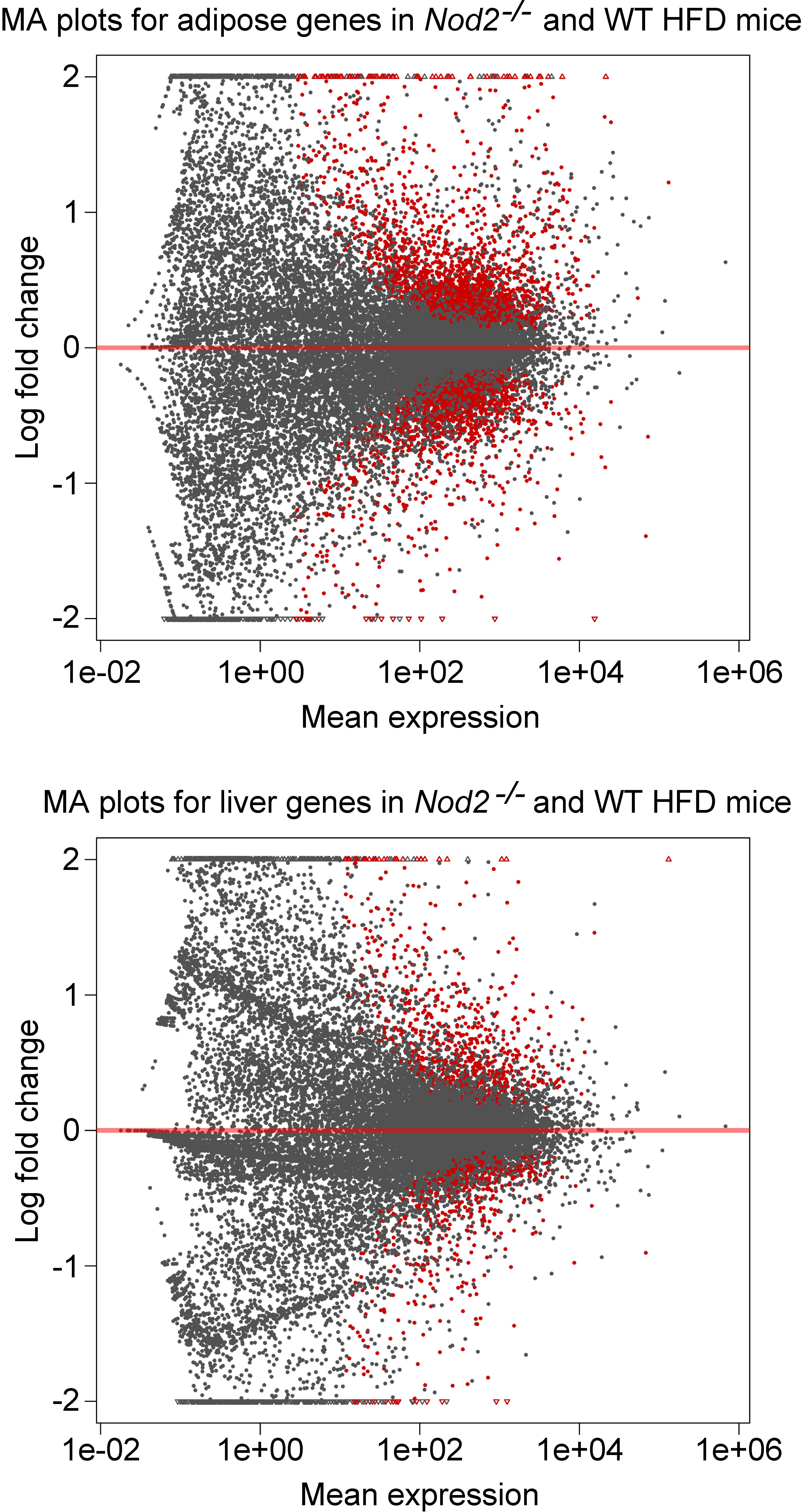


**Supplementary Figure S2. *Nod2*^-/-^ mice on HFD have many differentially expressed genes in the liver and adipose tissue compared with WT mice on HFD.** MA plots showing gene expression in the (A) liver and (B) adipose tissue of *Nod2*^-/-^ HFD mice relative to WT HFD mice. Each dot represents a gene. The Y-axis is the log2 fold change (*Nod2*^-/-^ HFD /WT HFD) and the X-axis is the average of the counts normalized by size factor. All genes falling on the red horizontal line intersecting at 0 on the Y-axis have a log2 ratio of zero (no change in gene expression). All the genes falling above 0 are up regulated in *Nod2*^-/-^, while those below 0 are down regulated. The genes colored red are significantly up or down regulated (significance defined at *P*≤0.05 at 5% FDR). The triangles represent genes that are differentially expressed over log2 fold change.


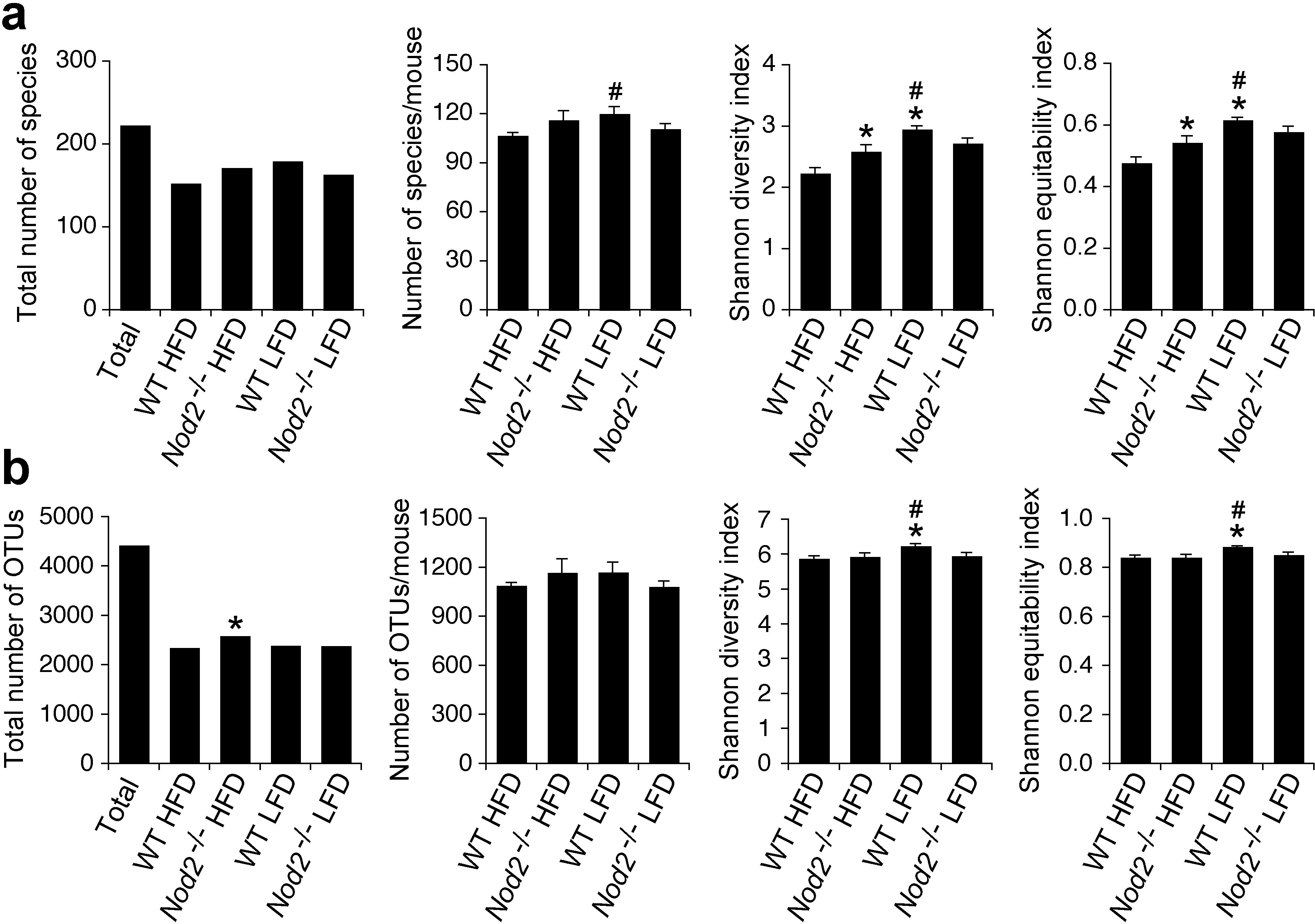


**Supplementary Figure S3. α-diversity in stool microbiota in WT HFD, *Nod2*^-/-^ HFD, WT LFD, and *Nod2*^-/-^ LFD mice.** (a) Total numbers of species, numbers of species/mouse, Shannon diversity index, and Shannon equitability index for bacterial species. (b) Total numbers of OTUs, numbers of OTUs/mouse, Shannon diversity index, and Shannon equitability index for bacterial OTUs. The results are means ± SEM or totals; *N* = 6 mice/group; **P*≤0.05 WT *versus* *Nod2*^-/-^ (on HFD or LFD); #*P*≤0.05 HFD *versus* LFD (for WT or *Nod2*^-/-^). The entire microbiome analysis data comparing bacterial diversity in the stools of WT and *Nod2*^-/-^ mice maintained on HFD and LFD have been deposited in NCBI SRA, accession No. SRP076031.


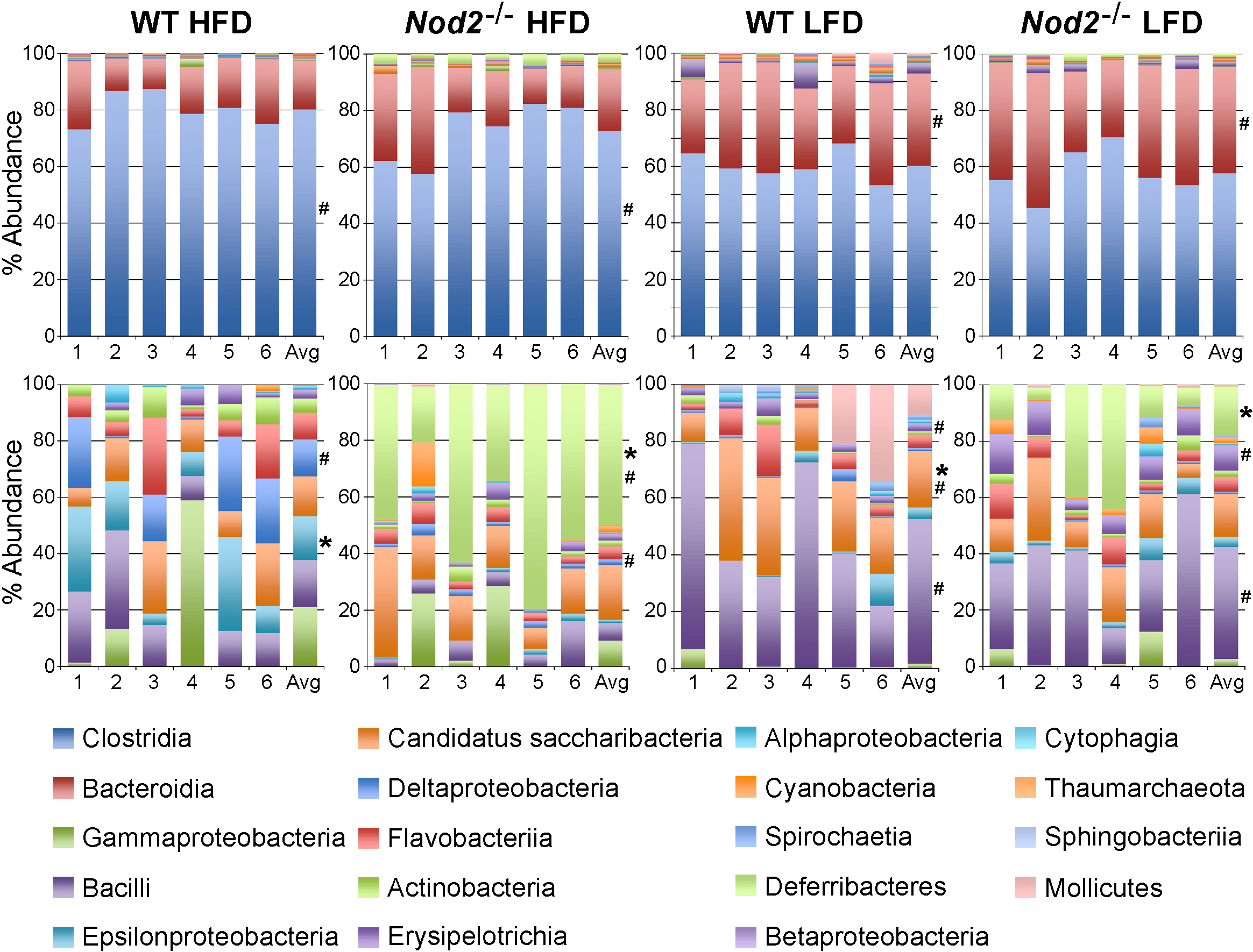


**Supplementary Figure S4. Class β-diversity in stool microbiota in WT HFD, *Nod2*^-/-^ HFD, WT LFD, and *Nod2*^-/-^ LFD mice.** Class abundance expressed as % of total stool microbiota is shown for individual mice (1-6) and an average (Avg) for all bacterial classes (top graphs) and for 17 less abundant classes expressed as % of total low abundant classes (excluding two most abundant classes, *Clostridia* and *Bacteroida*) (bottom graphs); *, classes with significantly (at *P*≤0.05) increased abundance in WT *versus* *Nod2*^-/-^ mice (on HFD or LFD); #, classes with significantly (at *P*≤0.05) increased abundance in HFD *versus* LFD groups (for WT or *Nod2*^-/-^ mice). The entire microbiome analysis data comparing bacterial diversity in the stools of WT and *Nod2*^-/-^ mice maintained on HFD and LFD have been deposited in NCBI SRA, accession No. SRP076031.
